# Decompression of Multimorbidity Along the Disease Trajectories of Diabetes Mellitus Patients

**DOI:** 10.3389/fphys.2020.612604

**Published:** 2021-01-05

**Authors:** Nils Haug, Johannes Sorger, Teresa Gisinger, Michael Gyimesi, Alexandra Kautzky-Willer, Stefan Thurner, Peter Klimek

**Affiliations:** ^1^Section for Science of Complex Systems, CeMSIIS, Medical University of Vienna, Vienna, Austria; ^2^Complexity Science Hub Vienna, Vienna, Austria; ^3^Department of Medicine III, Endocrinology and Metabolism, Medical University of Vienna, Vienna, Austria; ^4^Austrian National Public Health Institute, Vienna, Austria; ^5^Gender Institute, Gars am Kamp, Austria; ^6^IIASA, Laxenburg, Austria; ^7^Santa Fe Institute, Santa Fe, NM, United States

**Keywords:** comorbidity networks, disease trajectories, population aging, diabetes mellitus, machine learning, data visualization, multimorbidity

## Abstract

Multimorbidity, the presence of two or more diseases in a patient, is maybe the greatest health challenge for the aging populations of many high-income countries. One of the main drivers of multimorbidity is diabetes mellitus (DM) due to its large number of risk factors and complications. Yet, we currently have very limited understanding of how to quantify multimorbidity beyond a simple counting of diseases and thereby inform prevention and intervention strategies tailored to the needs of elderly DM patients. Here, we conceptualize multimorbidity as typical temporal progression patterns of multiple diseases, so-called trajectories, and develop a framework to perform a matched and sex-specific comparison between DM and non-diabetic patients. We find that these disease trajectories can be organized into a multi-level hierarchy in which DM patients progress from relatively healthy states with low mortality to high-mortality states characterized by cardiovascular diseases, chronic lower respiratory diseases, renal failure, and different combinations thereof. The same disease trajectories can be observed in non-diabetic patients, however, we find that DM patients typically progress at much higher rates along their trajectories. Comparing male and female DM patients, we find a general tendency that females progress faster toward high multimorbidity states than males, in particular along trajectories that involve obesity. Males, on the other hand, appear to progress faster in trajectories that combine heart diseases with cerebrovascular diseases. Our results show that prevention and efficient management of DM are key to achieve a compression of morbidity into higher patient ages. Multidisciplinary efforts involving clinicians as well as experts in machine learning and data visualization are needed to better understand the identified disease trajectories and thereby contribute to solving the current multimorbidity crisis in healthcare.

## 1. Introduction

Multimorbidity might well be one of the defining challenges for healthcare systems of high-income countries in the twenty-first century (Pearson-Stuttard et al., 2019). Fueled by an aging population, the percentage of people with two or more health conditions is steadily increasing which in turn drives morbidity and mortality (Soh et al., 2020; Whitty et al., 2020). One of the drivers of the rise of multimorbidity is diabetes mellitus (DM) due to its large number of physical and mental risk factors and complications (Chiang et al., 2020). For instance, heart disease and stroke are well-established complications of diabetes whereas overweight, hypertension, or tobacco use are known risk factors for diabetes (Xu et al., 2018). These complications and risk factors might interact with each other in ways that have yet to be understood. Consequently, we currently have limited knowledge of how multimorbidity develops in type 2 DM (T2D) patients over their life course.

One of the reasons for this knowledge gap is that it is not at all clear how to define multimorbidity (Nicholson et al., 2019). Conventionally, multimorbidity is often defined as the occurrence of two or more health conditions in a patient. However, prognosis and treatment of a patient depend on which diseases actually do co-occur (Steinhaeuser and Chawla, 2009; Chmiel et al., 2014). Recent research showed that patients describe disease progression patterns in the form of typical sequences of diseases over their life course; so-called disease trajectories (Jensen et al., 2014; Kannan et al., 2016; Giannoula et al., 2018; Haug et al., 2020). For instance, using electronic health records a typical trajectory toward T2D has been identified in which patients acquire hyperlipidemia, hypertension, impaired fasting glucose and finally DM, in that order (Oh et al., 2016). This and related research shows that multimorbidity is better understood in terms of typical disease trajectories, rather than a simple count of diagnoses.

The emerging field of network medicine (Barabàsi et al., 2011) has greatly helped our understanding of how such trajectories might look like based on EHR or medical claims data (Jensen et al., 2012). A number of works sought to identify pairs or groups of diseases with a statistical tendency to co-occur (Hidalgo et al., 2009; Park et al., 2009; Chmiel et al., 2014; Fotouhi et al., 2018). In brief, at younger age patients typically acquire fewer and physiologically closely related disorders (e.g., mental disorders that co-occur with substance abuse). The situation changes drastically for elderly patients with multi-factorial chronic disorders, including DM, that serve as risk factors for other diseases across the entire diagnostic spectrum (Chmiel et al., 2014). The existence of such disease networks is a direct consequence of the complex networks of physiological processes that underlie most diseases (Menche et al., 2015). For instance, the OMIM database currently lists around 30 genetic locations that are believed to have a causative impact on DM (Hamosh et al., 2005). Most of these genes are involved in other diseases as well, meaning that multimorbidity arises due to shared pathophysiological processes of the cooccurring diseases (Klimek et al., 2016). To factor such findings into improved medical strategies for early prognosis and treatment of patients, we have yet to understand how these trajectories vary between patients having a certain disease or not. That is, is a certain trajectory specific for patients that will acquire, say, DM later in their life or not?

The interrelatedness of many different diseases across the entire spectrum hints at an interconnectedness of the organ systems underlying the individual diseases. The emerging field of network physiology seeks to improve our understanding of how organ systems affect and interact with each other (Bashan et al., 2012; Bartsch et al., 2015). Physiological systems have non-stationary, intermittent, scale-invariant, and nonlinear behaviors. Therefore, their output dynamics transiently change in time with different physiologic states and under pathologic conditions (Ivanov et al., 2016). The dynamics of these complex systems are further complicated by various coupling and feedback interactions among different subsystems, which are not fully understood (Ivanov et al., 2016). Also in DM complex interactions of different organ systems could lead to specific comorbidities.

Here, we identify disease trajectories that are specific for DM patients over their entire life course using a hierarchical temporal clustering procedure. A cluster is given by a set of diseases that all patients in that cluster must have been diagnosed with so far, and another set that none of the patients has been diagnosed with so far. When patients acquire yet another disease, they might “move” to a new cluster, which can be described by transition probabilities between individual clusters. This induces a network structure in which the disease clusters can be represented as nodes and links indicating how likely a patient is to progress from one cluster to the next. Walks on this network that end at a specific cluster therefore encode the temporal information of all typical disease trajectories of patients with a corresponding set of diseases. By utilizing a hierarchical clustering algorithm (Chavent et al., 2007), we can further investigate these trajectories on multiple resolution levels.

By performing a matched comparison of the disease trajectories of DM patients with non-diabetic patients, we can identify those trajectories that are specific for DM. Due to the high number and hierarchical organization of disease clusters and their associated transition network, it is a considerable challenge to enable an exploration of these results for non-technical experts. We, therefore, also developed an interactive network visualization solution that allows, e.g., clinical practitioners, to perform controlled comparisons of DM and non-DM trajectories. We conclude this work by showing that our analysis and visualization system indeed recovers meaningful diabetic disease trajectories, from early risk factors to late-stage complications and show how our work can be used to generate new hypotheses on sex-specific differences of these trajectories.

## 2. Data and Methods

### 2.1. Study Population

Our study is based on a medical claims dataset covering approx. 45,000,000 hospital stays of about 9,000,000 Austrians over the time period from 1997 to 2014 (Haug et al., 2020). For each stay we consider patient age (5 year groups), sex and pseudo-ID, the main and side diagnoses associated with the stay, its date and type of discharge (e.g., normal release, transfer, or death). Diagnoses are provided as three digit ICD-10 codes. For the analysis we use 1,074 codes ranging from A00 to N99 and group them into 131 disease blocks as defined by the WHO.

The DM patient cohort consists of the 250,498 patients who (i) did not receive a diagnosis with ICD-10 code from A00–N99 between 1997 and 2002, (ii) and who did receive a diagnosis with ICD-10 code from E10 to E14 (DM) during the observation period from 2003 to 2014. The mean age of the patients at the beginning of the observation period is 60 y; 53% of the patients are male. Each cohort patient is matched with 2 non-diabetic control patients of the same age, sex, and region of origin, who (i) did not receive a diagnosis with ICD-10 code from A00 to N99 between 1997 and 2002, and (ii) who did not receive a diagnosis with ICD-10 code from E10 to E14 during the observation period.

### 2.2. Clustering

The health state of each patient at the end of each half-year within the observation period is represented by a binary row vector **v** = (*v*_1_, *v*_2_, …, *v*_*M*_) of length *M* = 131, where each dimension corresponds to one of the 131 ICD-10 code blocks considered (see Supplementary Material). For 1 ≤ *d* ≤ *M*, we have *v*_*d*_ = 1 if the patient has received a diagnosis from diagnosis block *d* until the end of that half-year and 0 else. The vectors representing the health states of each patient at the end of each half-year are then clustered using a divisive clustering algorithm called DIVCLUS-T, which was introduced in Chavent et al. (2007). The same clustering method has been used in Haug et al. (2020). This method defines a cluster by means of a set of inclusion and exclusion criteria (presence or absence of certain diagnoses) that all patients in the cluster have to fulfill. Each clustering step therefore divides an existing cluster by introducing an additional inclusion or exclusion criterion in a way that minimizes intra-cluster variance (Chavent et al., 2007). We use the elbow method to identify the optimal size of the set of disease clusters and hierarchically group these clusters into eleven so-called macro-clusters. The result is a multi-level hierarchy of a few macro-clusters (defined by distinct inclusion and exclusion criteria), each macro-cluster can further be divided into more fine-grained clusters using additional inclusion/exclusion criteria. More details on the clustering can be found in the Supplementary Material.

Note that the clustering is performed on the cohort of DM patients; control patients are subsequently assigned to the obtained clusters according to their diagnoses.

### 2.3. Matched Disease Trajectory Comparison

Disease trajectories of patients are described by sequences of clusters and transitions between them (Haug et al., 2020). If a patient of sex *s* and age group *a* is assigned to cluster *j* in one half-year period and to cluster *k* in the next half-year, we say the patient steps from *j* to *k*. For each sex and age group this gives rise to the cluster transition rate *q*_*s, a, k, j*_ (Haug et al., 2020), which is the probability that a patient in cluster *k* with age *a* and sex *s* steps to cluster *j* in each half-year. We compute the tensor *q*_*s, a, k, j*_ for two different patient populations. First, the tensor *q*^*DM*^ describes disease trajectories for DM patients. Second, the tensor *q*^*C*^ describes disease trajectories for their matched control group. The element-wise tensor difference, $RD_{s,a,k,j}=q_{s,a,k,j}^{DM}-q_{s,a,k,j}^{C}$, gives the risk difference between disease trajectories of diabetic and non-diabetic patients. The absolute risk difference *RD* measures whether DM patients are more (*RD* > 0) or less (*RD* < 0) likely to progress from one multimorbid health state (disease cluster) to the next compared to their non-diabetic controls. By taking the average of *RD* over all age groups *a* and/or sex *s*, we compute age- and/or sex-independent risk differences. To measure differences between disease trajectories of male and female DM patients, we consider sex risk difference $SRD_{a,k,j}=q_{\text{males},a,k,j}^{DM}-q_{\text{females},a,k,j}^{DM}$, which we again average over all age groups to obtain an age-independent sex risk difference.

### 2.4. Visualization Strategy

As a means to intuitively explore the results of our analysis, we have built an interactive exploration tool where users can perform controlled trajectory comparisons by themselves. The tool shows the composition of clusters and allows one to filter trajectory data by sex and age groups, to specify thresholds for transition probabilities and to re-arrange the network layout. Two groups of patients (specified by DM or non-DM and male, female, or both) can be compared with each other and the results can be downloaded for further analysis. A detailed description is given in section 3.6.

## 3. Results

### 3.1. Baseline Characteristics

We identify 250,498 DM patients in our study population and 500, 996 in the matched control group. Of the study population, 116, 758 (47%) are female. The median age of the population at the beginning of the observation period is 67 (54–77) for females and 58 (48–67) for males; values in brackets give the range from lower to upper quartiles. The median number of diagnoses per patient is 8 (5–12) for females and 8 (4–11) for males in the DM group, whereas in the control group we find 3 (1–7) for females and 3 (1–6) for males.

### 3.2. Multi-Level Clusters for Multimorbid Health States

We identify 128 disease clusters that can be grouped into 11 macro-clusters. Each cluster and macro-cluster is described by a set of diagnoses that all patients in that cluster must have (inclusion criteria) and diagnoses that none of the patients have (exclusion criteria). For the 11 macro-clusters these conditions are reported in Supplementary Tables 1–11. Each of these macro-clusters contains a variable number of clusters listed with their inclusion and exclusion criteria in Supplementary Tables 12–139.

To give an overview of these results, we give a list of all macro-clusters with diagnoses that appear at least once as inclusion criteria in a sub-cluster of that macro-cluster in Table 1. The macro-clusters are labeled with short names and roughly correspond to the inclusion criteria of the most populated cluster in each macro-cluster. The clusters are hierarchically ordered in a way such that macro-clusters with a higher ID tend to have more inclusion criteria, i.e., the patients in that cluster typically have more diagnoses.

**Table 1:** For each macro cluster, the table lists the diagnosis blocks which appear as most frequent inclusion criteria in at least one cluster belonging to that macro cluster, along with a descriptive short name and mean patient age.

**ID**	**Short name**	**Age**	**Inclusion criteria**
0	Misc	61y	Other diseases of upper respiratory tract; Intestinal infectious diseases; Disorders of choroid and retina; Other diseases of urinary system; Organic, including symptomatic, mental disorders; Mental and behavioral disorders due to psychoactive substance use; Arthropathies; Dorsopathies; Soft tissue disorders
1	Metabolic disorders	63 y	Diseases of liver; Diseases of arteries, arterioles and capillaries; Arthropathies; Ischaemic heart diseases; Metabolic disorders; Dorsopathies
2	Disorders of lens	74 y	Glaucoma; Disorders of choroid and retina; Disorders of vitreous body and globe; Arthropathies; Disorders of lens
3	Cerebrovascular diseases	75 y	Cerebrovascular diseases; Episodic and paroxysmal disorders; Diseases of arteries, arterioles and capillaries; Disorders of lens; Other degenerative diseases of the nervous system
4	Malignant neoplasms	73 y	Neoplasms of uncertain or unknown behavior; Other diseases of urinary system; Malignant neoplasms; Disorders of lens; Chronic lower respiratory diseases
5	Obesity and other hyperalimentation	62 y	Episodic and paroxysmal disorders; Mental and behavioral disorders due to psychoactive substance use; Obesity and other hyperalimentation; Diseases of liver; Noninflammatory disorders of female genital tract; Ischaemic heart diseases; Arthropathies; Metabolic disorders; Dorsopathies
6	Diseases of oesophagos, stomach and duodenum	68 y	Cerebrovascular diseases; Other diseases of intestines; Mental and behavioral disorders due to psychoactive substance use; Diseases of esophagus, stomach and duodenum; Diseases of liver; Malignant neoplasms; Dorsopathies; Disorders of gallbladder, biliary tract and pancreas; Hernia
7	Other form of heart disease	76 y	Other diseases of intestines; Organic, including symptomatic, mental disorders; Malignant neoplasms; Other degenerative diseases of the nervous system; Ischaemic heart diseases; Disorders of lens; Metabolic disorders; Obesity and other hyperalimentation; Other forms of heart disease
8	Other form of heart disease + Chronic lower respiratory dis.	75 y	Dorsopathies; Influenza and pneumonia; Other diseases of the respiratory system; Obesity and other hyperalimentation; Chronic lower respiratory diseases; Other forms of heart disease
9	Other form of heart disease + Cerebrovascular diseases	80 y	Cerebrovascular diseases; Episodic and paroxysmal disorders; Organic, including symptomatic, mental disorders; Other degenerative diseases of the nervous system; Diseases of arteries, arterioles and capillaries; Other diseases of urinary system; Ischaemic heart diseases; Disorders of lens; Dorsopathies; Other forms of heart disease
10	Other form of heart disease + Renal failure	80 y	Osteopathies and chondropathies; Renal failure; Other forms of heart disease; Aplastic and other anaemias; Organic, including symptomatic, mental disorders; Diseases of arteries, arterioles and capillaries; Other diseases of urinary system; Other disorders of the skin and subcutaneous tissue; Disorders of lens; Chronic lower respiratory diseases

In brief, we first find a macro-cluster of miscellaneous diseases, cluster 0. We then have a series of macro-clusters characterized by metabolic disorders, disorders of lens, cerebrovascular diseases, malignant neoplasms, obesity, diseases of esophagus, and heart diseases, respectively. Finally, we have three macro-clusters in heart diseases combined with chronic lower respiratory diseases, cerebrovascular diseases, and renal failure, respectively.

### 3.3. Disease Trajectories

**Figure 1:**
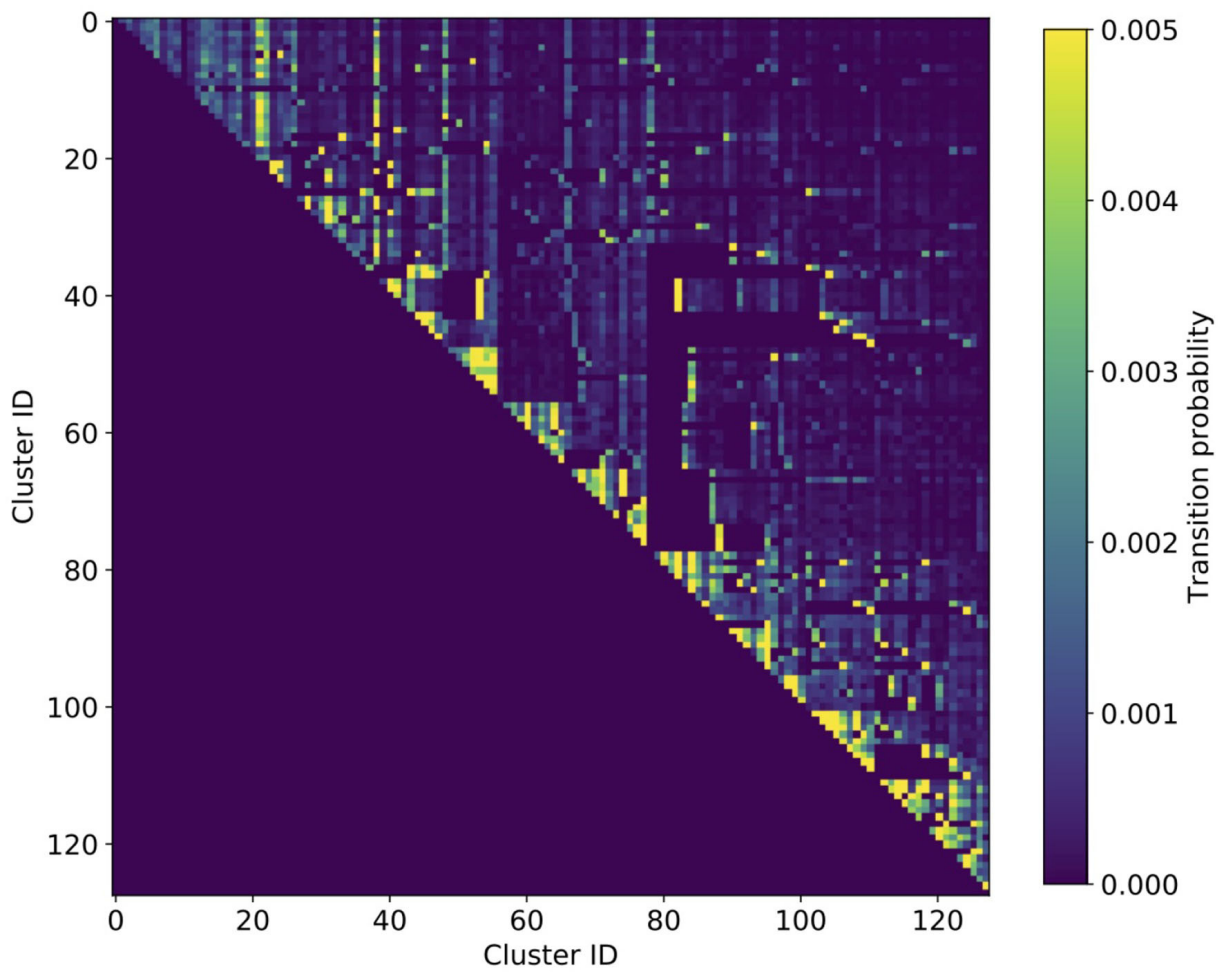
Heatmap of cluster transition rates aggregated over age and sex. The transition matrix is of triangular shape, meaning that there is a by construction hierarchical order of how patients progress in their multimorbid health states. The multi-level hierarchy is clearly discernible by the triangular blocks with increased transition rates along the diagonal, the macro-clusters.

Results for the transition rates between the 128 identified clusters are shown in Figure 1 as a heat map, aggregated over age, and sex. First, we note the by construction upper-triangular shape of the matrix indicating that patients always step to a cluster with a higher ID than their current one. Second, we can clearly see the macro-clusters as blocks along the diagonal with comparably high transition rates. This means that most steps (health state transitions) take place within the same macro-cluster and steps from one macro-cluster to another occur more seldomly.

**Figure 2:**
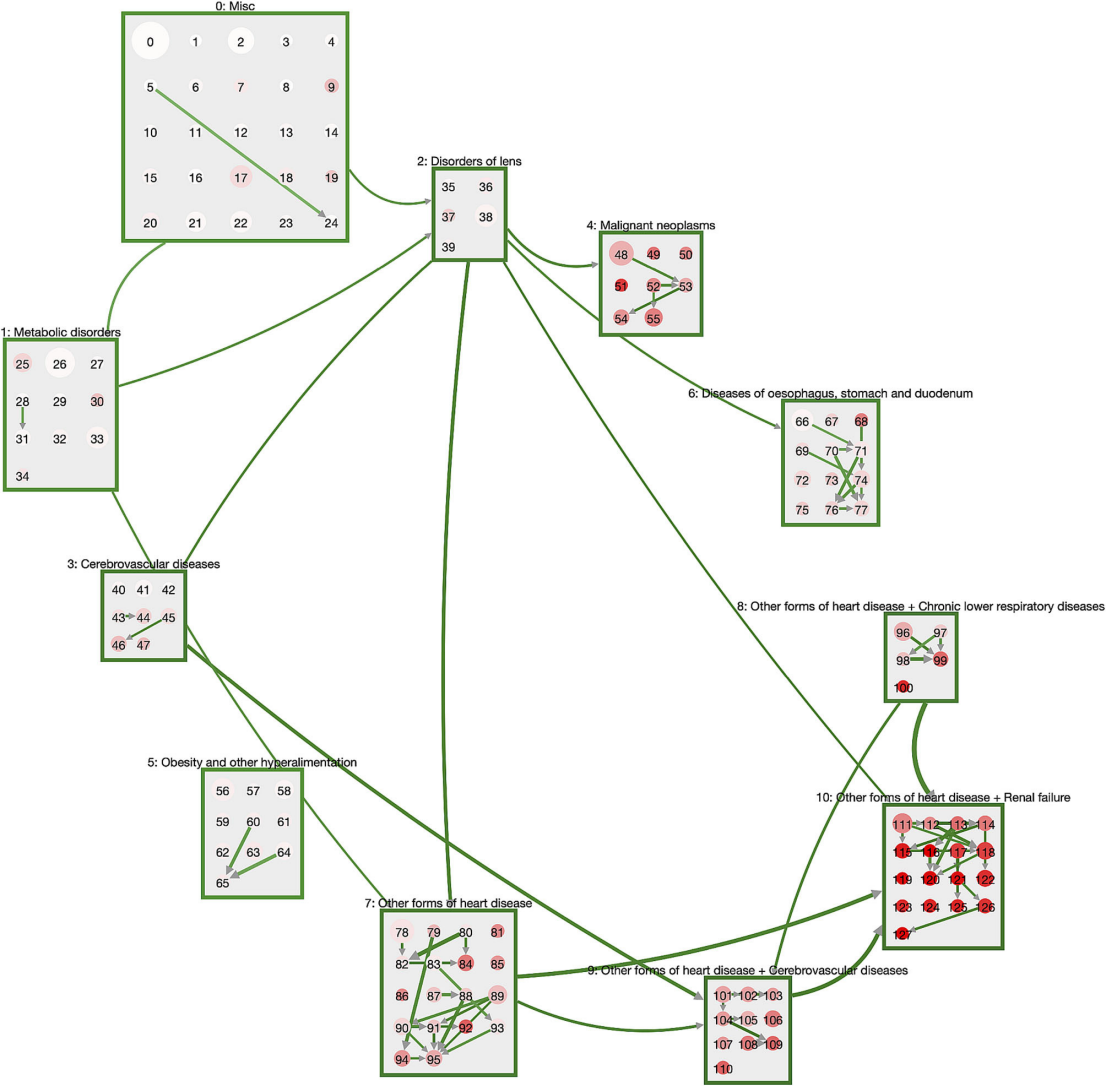
Trajectories of DM patients. The macro-clusters are shown as green squares containing a variable number of disease clusters. Cluster size is proportional to the number of patients in that cluster; color gives mortality (the more intense the red, the higher the mortality). We show links for transitions between macro-clusters or between clusters of the same macro-cluster with a weight (transition rate) of at least 0.007. DM patients typically start in the “Misc” cluster and progress via metabolic disorders and eye diseases toward heart diseases that combine with respiratory and cerebrovascular diseases, as well as renal failure.

In Figure 2, we show the network of disease trajectories for DM patients, filtered to links with a minimum weight (transition rate) of 0.007. Node size gives the number of patients in the cluster, color describes the in-hospital mortality in the cluster. Again, we note the hierarchical order of macro-clusters. In general, there is a clear trend that the higher the cluster ID, the higher the mortality. Highest mortality is found in the macro-cluster for patients with heart diseases and renal failure, where the patients also acquired respiratory or cerebrovascular diseases before stepping into that macro-cluster. An exception to this general trend is the macro-cluster of malignant neoplasms, where mortality is particularly high in cluster 51 (see Supplementary Table 63) when cancer combines with aplastic anemia.

The general pattern of disease trajectories in DM patients can be described as follows. Patients start their journey in the macro-cluster “Misc” which is defined by exclusion criteria for neoplasms, obesity, metabolic disorders, disorders of lens, heart diseases, cerebrovascular diseases and diseases of the esophagus, stomach and duodenum. Loosely speaking, patients are at their “healthiest” in this cluster. For instance, the disease cluster 0 consists solely of exclusion criteria and has no inclusion criteria (Supplementary Table 12). Next, they often acquire either metabolic disorders or disorders of the lens. These diseases are typically followed by neoplasms, cerebrovascular diseases, or diseases of oseophagus, stomach, and duodenum. The subsequent stage is the acquisition of heart diseases, particularly if diseases from the obesity cluster are also present. From the combination of diabetes with heart diseases we see the development of highly multimorbid patient states with additional diagnoses of, e.g., stroke (cluster 105, Supplementary Table 117), chronic lower respiratory diseases coupled with pneumonia (cluster 99, Supplementary Table 111) or renal failure (cluster 113, Supplementary Table 125).

The dynamics within the individual macro-clusters is typically of the following form. Patients “start out” in a cluster that has the same inclusion criteria as the corresponding macro-cluster, e.g., cluster 111 (Supplementary Table 123) that contains patients with heart diseases and renal failure. With a transition rate of 0.0074 patients step into cluster 118 (Supplementary Table 130) where they are additionally diagnosed with diseases of the arteries and disorders of the skin and subcutaneous tissue. Other patients of cluster 111 acquire organic mental diseases (such as dementia) and other degenerative diseases of the nervous system and step with a transition rate of 0.005 to cluster 122 (Supplementary Table 134). In summary, we see that all patients in a macro-cluster share a certain set of diabetic comorbidities and then branch into different additional comorbidities within the same macro-cluster. Occasionally we observe similar trajectories within different macro-clusters. For instance, cluster 110 (Supplementary Table 122) has the same inclusion criteria as cluster 122, with cerebrovascular diseases instead of renal failure.

### 3.4. Comparing Trajectories of DM Patients With Their Non-diabetic Controls

**Figure 3:**
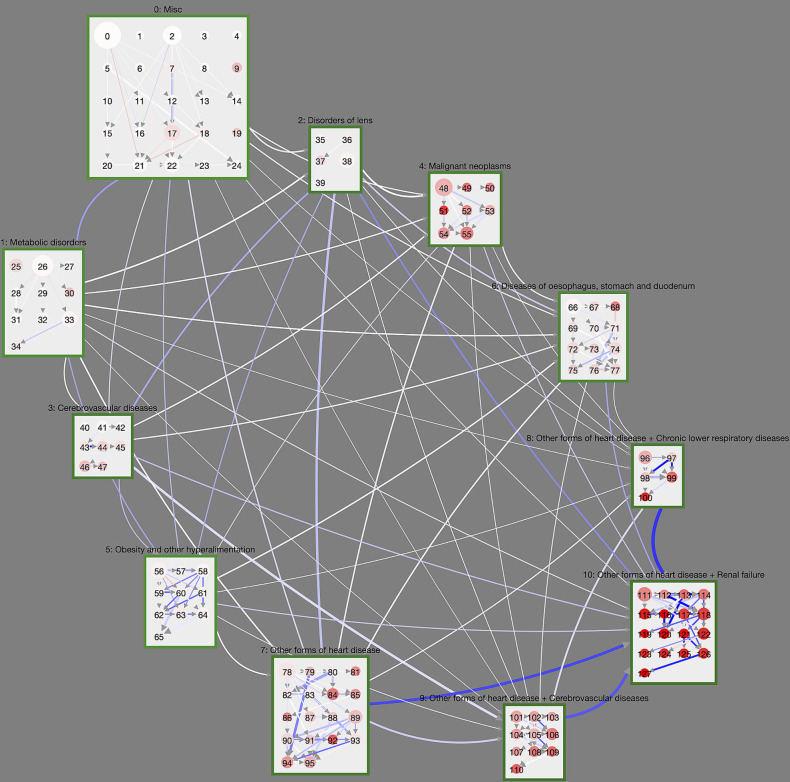
Controlled trajectory comparison of DM patients with their non-diabetic controls. We show the unfiltered disease trajectory network with weights giving the difference in cluster transition rates, *RD*. The more intense the blue (red), the more (less) frequent is the corresponding cluster transition in DM patients compared to non-DM patients. Almost all cluster transitions are overrepresented in DM patients, meaning that diabetes patients overall progress within shorter time-periods toward highly multimorbid health states. This is particularly the case for macro-clusters with heart diseases.

A graphical summary of the results of a matched trajectory comparison between diabetes patients and their non-diabetic controls is shown in Figure 3. We show cluster and macro-clusters as in Figure 2, however, here link weights represent the absolute risk difference *RD* of the corresponding cluster transition. In principle, we show transitions that are more frequent for DM patients as blue and those more frequent for non-DM patients in red. However, the network is clearly dominated by blue links indicating that DM patients have higher rates for almost all cluster transitions compared to non-diabetic controls. This means that diabetes patients progress faster along their disease trajectories.

We observe particularly large differences for macro-clusters that include heart diseases, i.e., that have a cluster ID of 7 or higher. For instance, patients with heart diseases in cluster 7 step at rate 0.016 to cluster 10 (heart diseases and renal failure) if they have DM, whereas the rate for the controls is 0.0084. From cluster 8 (heart diseases and chronic lower respiratory diseases) DM patients step at rate 0.020 to cluster 10; non-diabetes patients at rate 0.012. Similarly, from macro-cluster 9 (heart diseases and cerebrovascular diseases) DM patients have a rate of 0.016 for transitions to cluster 10; for non-DM patients the rate is 0.0097.

Considering the dynamics within macro-clusters, there are clearly discernible clusters with concentrated DM trajectories.

These can be seen in Figure 3 as clusters with many incoming and/or outgoing links with a high *RD* (blue color). This includes cluster 94 (Supplementary Table 106), which contains ischaemic heart diseases, other forms of heart diseases and diseases of arteries, and “attracts” patients from its surrounding clusters much stronger if they have DM. There is an abundance of DM trajectories leaving from cluster 58 (Supplementary Table 70, obesity and metabolic disorders) and leading to clusters that also contain ischaemic heart diseases (59, Supplementary Table 71) or episodic and paroxysmal disorders (62, Supplementary Table 74).

### 3.5. Comparing Trajectories of Male and Female DM Patients

**Figure 4:**
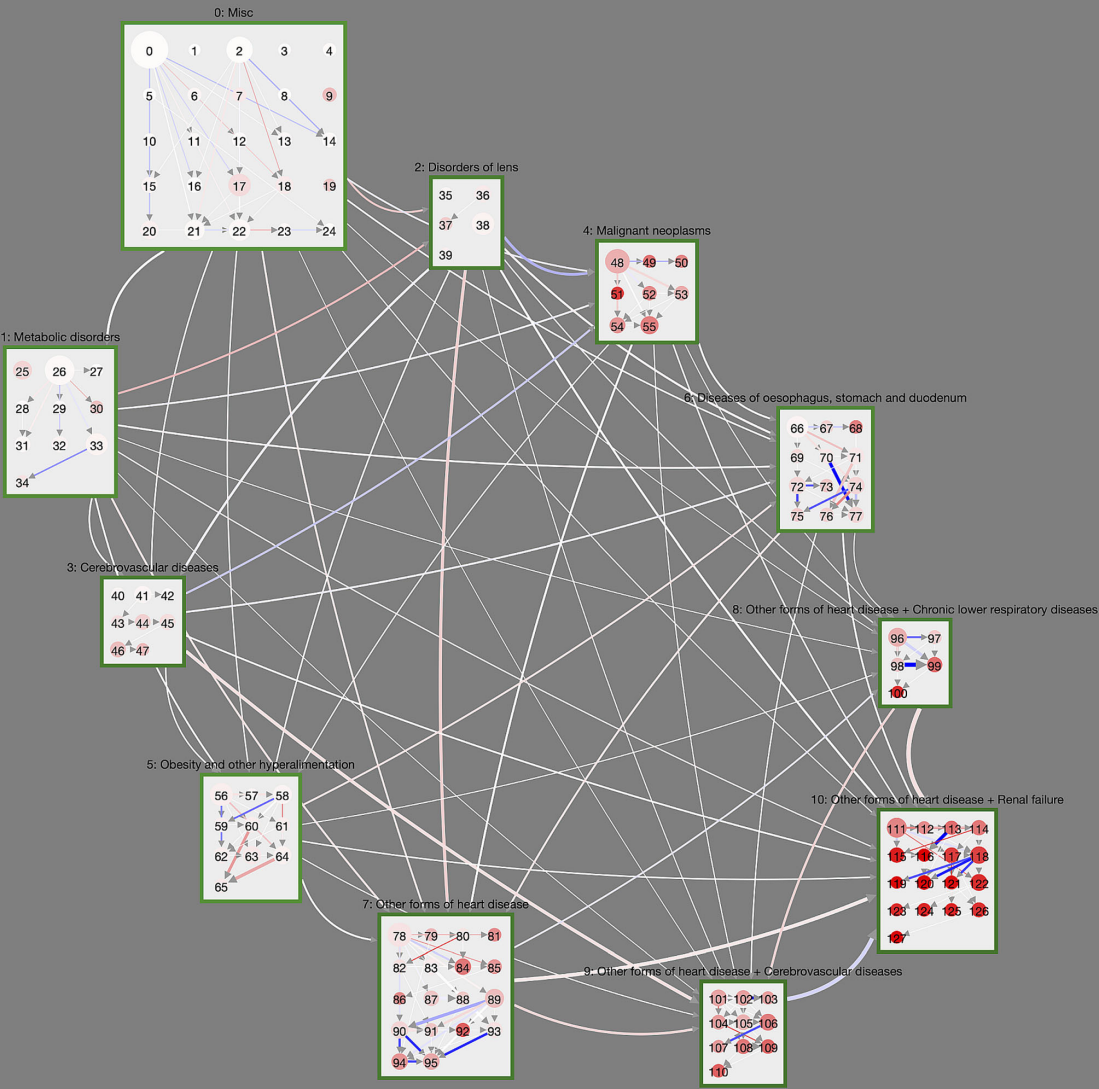
Controlled trajectory comparison of male and female DM patients. Here, link weights are given by the age-averaged sex risk difference *SRD*. Blue (red) links indicate that the corresponding cluster transition is more (less) frequently observed in trajectories of male DM patients compared to female DM patients. We see that certain cluster transitions in the highly multimorbid macro-cluster 10 is dominated by male DM patients, whereas some trajectories in the obesity macro-cluster are dominated by female patients.

Figure 4 compares the trajectories of male and female DM trajectories. The cluster layout is again taken from Figure 2; link weights now indicate the sex risk difference *SRD* for the individual cluster transitions. Transitions that are dominated by male DM patients (*SRD* > 0) are shown in blue, female dominated ones in red (*SRD* < 0). Overall, we see that there is a clear tendency for most transitions between macro-clusters to be slightly overrepresented in female DM trajectories. One exception to this general trend are malignant neoplasms, which have a stronger in-flow of male DM trajectories. For reference, we show the trajectory network for the sex risk differences computed in the control group, instead of the DM group, in Supplementary Figure 1. There we see the same general tendency of more female inter-macro-cluster transitions but more male transitions toward malignant neoplasms. This means that these sex-specific features of the macro-cluster transitions are not specific to the DM population and rather describe general sex differences in the population.

On the level of individual clusters, we observe a couple of substantial sex risk differences between male and female DM patients that cannot be observed in the non-diabetic control. For male DM patients this includes trajectories toward cluster 95 (Supplementary Table 107) with ischaemic heart diseases, other heart diseases and diseases of the intestines and trajectories originating from cluster 118 (Supplementary Table 130, diseases of the heart, arteries, skin, and renal failure). There are also male-dominated DM trajectories that combine heart diseases, cerebrovascular diseases, diseases of the arteries and dorsopathies (toward cluster 107, Supplementary Table 119), which cannot be observed in non-diabetic controls.

Females trajectories are particularly over-represented for DM patients in the obesity macro-cluster. These trajectories involve cluster 60 (Supplementary Table 72) where obesity occurs with arthropathies and eventually combines with dorsopathies toward cluster 65 (Supplementary Table 77).

### 3.6. Visual Exploration of Results

In order to provide medical practitioners with an intuitive way to gain insights into our analysis results, we developed the *Disease Net Viewer*^1^, an online interactive visualization tool. The viewer gives users an overview of the cluster distributions within the hierarchy (see Figure 2), as well as the option to explore details, e.g., on in- or exclusion criteria, and the possibility to compare disease trajectories as shown in Figures 3, 4.

To convey the cluster hierarchy and disease trajectories, our tool displays clusters and their interrelations within a hierarchical node-link diagram. Clusters are represented as nodes that inform users about the cluster size (i.e., the number of patients within a cluster) and the cluster mortality. The cluster size is thereby represented in the node size. Cluster mortality is represented in the node color, by a gradient between white (low mortality) and red (high mortality). The detailed values can be accessed in a tooltip on mouse-over. Macro-clusters describing a set of clusters that share common inclusion and exclusion criteria, are represented by compound nodes that encompass all associated cluster nodes. The mean age of patients within a macro-cluster can be accessed on mouse-over.

The probability of a patient transitioning from one (macro-)cluster to another, is conveyed by the link between a pair of cluster nodes. The link's thickness and color-intensity thereby informs a user about the transition probability. The actual probability value can be accessed via tooltip. The minimum probability threshold for links to be included in the visualization can be adjusted by the user.

For a clean overview of clusters and their interrelations, we position macro clusters in an elliptical layout that highlights the flow of patients between clusters. We thereby place macro cluster 0 (lowest mortality) in the top left corner and macro cluster 10 (highest mortality) in the bottom right corner. Low-level clusters are placed in sequential order on a rectangular grid within their encompassing compound nodes. To further facilitate the exploration of the disease network topology, the selection of a cluster node visually highlights all its down-stream neighbor clusters.

**Figure 5:**
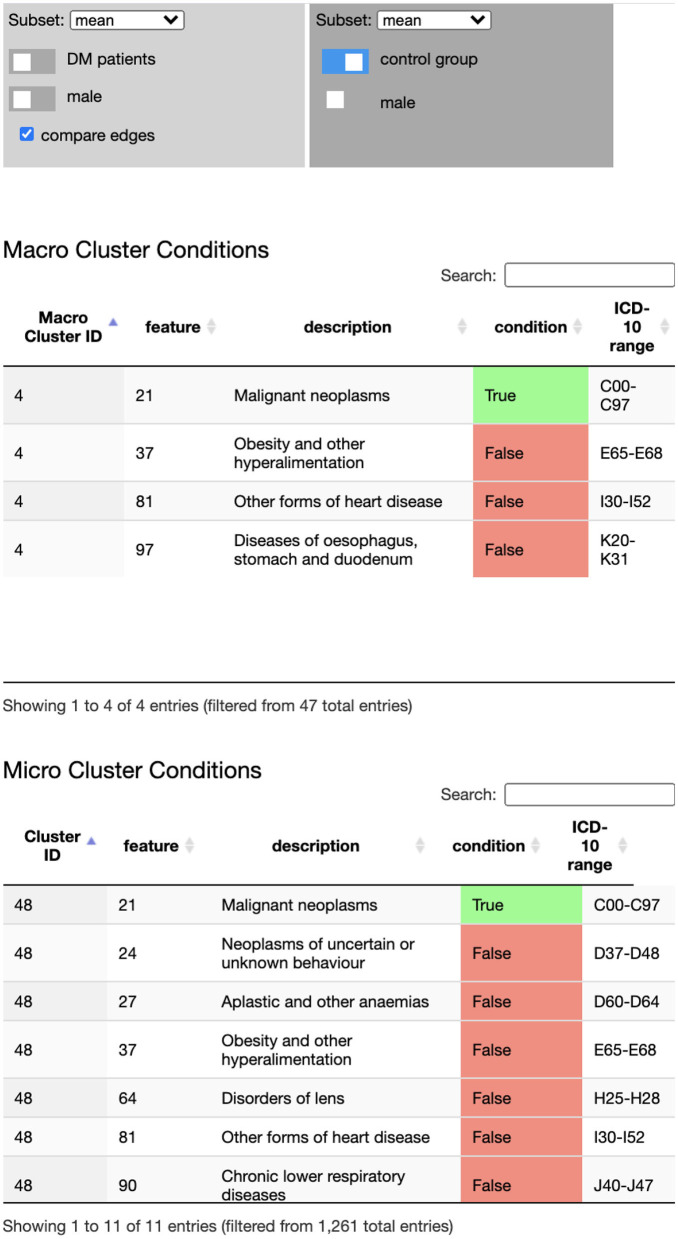
The Disease Net Viewer side bar hosts the data selection (and comparison) panel **(Top)**, the macro cluster table **(Middle)**, and the cluster table **(Bottom)**. The data selection panel controls, which data (sub)set is displayed in the network view's node link representation: average, sex specific average, age groups, DM cohort/control group, male/female. The cluster tables display inclusion and exclusion diagnose criteria for cluster memberships.

Detailed information on cluster conditions, i.e., their inclusion and exclusion criteria, is provided in the cluster and macro cluster tables (see Figure 5), providing the criterion (diagnose) description and the ICD-10 range. The tables and the node-link representation are linked to support more efficient exploration of the underlying data: browsing the table highlights the cluster node that corresponds to the currently selected table row. Selecting a node in the network, filters the table content to display the matching cluster criteria. The table content can also be searched, e.g., to find specific clusters that include or exclude certain ICD-10 codes.

Finally, the Disease Net Viewer enables users to compare trajectory probabilities between two different data (sub)sets; for instance between male and female DM patients, different age groups, or the DM cohort and the control group. The difference in probabilities is thereby visually highlighted in each transition link's color: a negative difference is indicated by a color gradient from white toward red; and a positive difference by a color gradient toward blue. The exact difference can again be accessed in the link's tooltip.

## 4. Discussion

While the challenge of multimorbidity in an aging population has long been recognized in medical research, it is not clear yet how we can properly quantify multimorbid health states beyond a simple counting of the number of diseases. Consequently, we currently have limited understanding on how multimorbidity in DM patients differs from the general population in terms of disease trajectories and how they lead to highly multimorbid health states and high mortality. This requires not only an adequate formal framework to quantify multimorbidity in patients, but also a way to compare different patient populations.

To fill this current knowledge gap, we built on a recently developed framework to quantify disease trajectories in patients (Haug et al., 2020). The main idea here is that diseases do not appear randomly and independent in patients, but in specific temporal patterns that can be identified using a hierarchical clustering approach. This procedure leads to disease clusters that can easily be interpreted by non-technical experts in terms of inclusion and exclusion criteria for certain diseases. Moreover, the hierarchical ordering of clusters encodes the disease history of patients within a cluster to some extent, as there are logical constraints on which cluster transitions are possible (e.g., it is not possible to step from a cluster where a certain disease is an inclusion criterion to another cluster where the same disease is an exclusion criterion).

Here, we extended this disease trajectory framework in a way that allows for matched comparisons of the trajectories of different patient populations. In particular, we considered DM patients and compared them to a cohort of matched non-diabetic controls. Furthermore, we compared the trajectories of male and female DM patients. To identify those sex differences that are specific for DM patients, we compared these results to sex differences in the matched control group.

We showed that the trajectories of DM patients can be organized in a multi-level hierarchy of macro-clusters and more fine-grained disease clusters. Thereby DM patients start their trajectories in a cluster with no or very few inclusion criteria for diseases at an age of around 61 y. As they age, they progress from early diabetic complications and comorbidities (metabolic disorders, eye disorders) to more multimorbid health states characterized by cardiovascular diseases in combination with cerebrovascular diseases, respiratory diseases, and/or renal failure.

By comparing the trajectories of DM patients to those of their non-diabetic controls, we find that DM patients show in general substantially higher rates at which they transition between clusters. This means that the progression from relatively healthy clusters of low mortality to highly multimorbid clusters with high mortality occurs at a much faster pace in DM patients compared to their non-diabetic controls. In this sense, DM accelerates the unhealthy aging process substantially. This acceleration is particularly strong for trajectories that involve heart diseases. This finding clearly suggests that DM (or its absence) plays an important role in the so-called compression of morbidity, i.e., the hypothesis that healthy aging can be achieved by compressing the burden of lifetime illness into a shorter period of time before death (Fries, 1980). We find that patients without DM might “end up” in the same highly multimorbid high-mortality disease clusters as DM patients, but they move toward these clusters at a much slower rate. In this sense, (cardiovascular) morbidity of non-diabetic patients is compressed toward higher ages.

The complex and highly multimorbid disease trajectories (spanning health conditions across the entire diagnostic spectrum) we identified in this work strongly suggest that DM with its precursors and complications arises due to cascading failures in the network of interconnected and interacting organ systems that make up the human organism (Ivanov and Bartsch, 2014). The topological structure of these networks of organ system is yet to be understood in particular from a mathematical and modeling point of view (Ivanov et al., 2016). Our novel statistical approach for matched cohort comparisons of disease trajectories might further the network physiology agenda by enabling the generation of new hypotheses regarding how such networks might differ from each other in different patient cohorts.

In particular, our novel framework also allowed us to compare the trajectories of male and female DM patients which can now be interpreted in terms of how the observed differences might be related to physiological differences. We find a tendency that females have overall higher cluster transition rates than males. Given the higher life expectancy of females compared to males, this finding is maybe surprising. However, recent research repeatedly showed that diabetes is a stronger risk factor for a number of complications in females compared to males. Females with DM had a higher mortality rate for cardiovascular diseases, coronary heart disease and stroke compared to male DM patients (Wang et al., 2019), even though studies claim that estrogen has a protective effect against DM (Tramunt et al., 2020). However, the general population of DM patients is over 60 years old and therefore already underwent menopause. The decrease of estrogen in postmenopausal women is known to increase the risk of impaired glucose tolerance, obesity and insulin resistance (Tramunt et al., 2020). That could be the reason why female DM patients show a faster progression along their trajectories in particular for disease clusters that involve obesity in combination with arthropathies and/or dorsopathies. Two key factors of developing diabetes and progression to its complications are overweight and obesity. Studies reported that women had a higher average BMI than men when first diagnosed with diabetes (Logue et al., 2011; Paul et al., 2012). Therefore, women might have more complications and diseases related to obesity. However, also social factors could play a role. For instance, a US study revealed that women were less likely to adhere to antidiabetic medication compared to men (Kirkman et al., 2015). Further it has been demonstrated that women with diabetes still receive less guideline-recommended care than men, even in the most developed countries (Peters and Woodward, 2018).

The investigation of multimorbidity and its associated trajectories also requires the development of novel tools to visualize and communicate its properties. In the scope of this work, we thus developed an interactive online viewer for illustrating cluster compositions and trajectories in an intuitive way for non-technical audiences, available under the address https://csh.ac.at/vis/diseasenet_viewer/. The visual interface currently is limited to representing the results of our analysis. In the future, however, it might be interesting to increase the tool's capabilities, in order to also support data scientists and medical professionals alike already during the data analysis process. To this end, the extension of our tool to support interactive clustering and statistical evaluation of the raw patient data will be of interest. Furthermore, at the moment our tool is only capable to compare DM patients with the general population though it is straight-forward to extend the analysis to focus on cohorts defined by other diagnoses (combinations) than DM and compare those with the general population.

With the use of medical claims data come a number of limitations concerning our study. Diagnoses might be misclassified or underrepresented particularly if they are not relevant for billing purposes. As diagnoses are only available for hospital care, also health problems that are typically treated in outpatient settings will likely be underreported in the data. Further, our study does not include information on socio-economic (education, family history, migration status, socio-economic status, etc.) and clinical parameters (BMI, HbA1c, etc.). Information on diagnoses made before the start of the observation period are also not reported in the data.

In conclusion, we have presented a novel methodological framework to perform matched comparisons between different patient populations in terms of their disease trajectories. We find that early prevention and treatment of DM is a key factor to enable healthy aging by compressing cardiovascular diseases, respiratory diseases, cerebrovascular diseases, renal failure, and a combination thereof toward higher ages. Further interdisciplinary efforts that bring clinical knowledge together with machine learning and high-dimensional data visualization are necessary to better understand how to treat an aging population.

## Data Availability Statement

This study was conducted using a pseudonymized research dataset only accessible for selected research partners under strict data protection regulations. Requests to access these datasets should be directed to Peter Klimek, klimek@csh.ac.at.

## Ethics Statement

Ethical review and approval was not required for the study on human participants in accordance with the local legislation and institutional requirements. Written informed consent from the participants' legal guardian/next of kin was not required to participate in this study in accordance with the national legislation and the institutional requirements.

## Author Contributions

NH, JS, and PK conceived the study design. MG acquired and contributed to analyzing the data, NH analyzed the data. JS developed the data visualization tool. PK wrote the first draft of the manuscript. NH, JS, and TG contributed to the writing. NH, JS, TG, AK-W, ST and PK reviewed and edited the manuscript.

## Conflict of Interest

The authors declare that the research was conducted in the absence of any commercial or financial relationships that could be construed as a potential conflict of interest.

## Footnotes

^1^https://csh.ac.at/vis/diseasenet_viewer/
