# Supplementary material for: Decompression of Multimorbidity Along the Disease Trajectories of Diabetes Mellitus Patients
Source: Front Physiol. 2021 Jan 5;11:612604. doi: 10.3389/fphys.2020.612604 (PMC7813935; doi:10.3389/fphys.2020.612604)
Supplement: Supplementary file 1 [file Data_Sheet_1.PDF]

Supplementary Information

1 SUPPLEMENTARY TEXT

1.1 Details on on the clustering algorithm

In this work we use a divisive hierarchical clustering algorithm called DIVCLUS-T (?). The application of this algorithm to diagnosis data was already described in previous work of us (Haug et al., 2020). The algorithm is closely related to well established clustering algorithms that rely on Ward's minimum variance method or the k-means algorithm. In particular, DIVCLUS-T optimizes the same objective function as these two clustering methods but emphasises interpretability of the results. The criteria, which DIVCLUS-T uses to separate clusters, are in the form of inclusion or exclusion conditions for diagnoses in a given cluster, which can also be readily interpreted by non-technical experts. In the description of the algorithm we follow Haug et al. (2020).

The diagnosis data is prepared as input for the algorithm in the following way. The data matrix $\mathbf{X} \in \{0, 1\}^{M\times N}$, records M observations (patients) of N different features (diagnoses). The (i, j) element of $\mathbf{X}$ is denoted by $X_{i,j}$, where $1 \leq i \leq M$ and $1 \leq j \leq N$. Furthermore, the data vector for one particular patient $1 \leq i \leq M$ we denote by $\mathbf{x}^{(i)} = (X_{i,1}, \ldots, X_{i,N})$. One row of $\mathbf{X}$ therefore encodes the diagnosis information for one specific patient at an instance in time. Column correspond to blocks of diagnoses. We set $X_{i,j} = 1$ if patient i has been diagnosed with diabetes before, 0 otherwise.

The algorithm constructs the clusters as follows. The algorithm is initiated with a root node, denoted by S_0. Iteratively, it constructs a binary tree in which each node k represents s specific subset S_k of the set of all observations $S_0 = \{1, \ldots, M\}$. Note that $S_k \supset S_j$ holds if and only if node k is an ancestor of node j. The subsets corresponding to the leaf nodes of any binary subtree of the full tree which contains the root node of the full tree form a partition of the total set of observations. We now associate with each node k a vector $\mathbf{p}^{(k)} = (p_1^{(k)}, \ldots, p_N^{(k)}) \in [0, 1]^N$, defined as

$$\mathbf{p}^{(k)} = 1|S_k| \sum_{i\in S_k} \mathbf{x}^{(i)}. \tag{S1}$$

That is, $\mathbf{p}^{(k)}$ is nothing but the centroid of the $|S_k|$ binary vectors represented by node k. The probability $p_j^{(k)}$ gives the chance that a randomly selected observation assigned to cluster S_k has feature j. For this clustering algorithm one can define the inertia of a node k as

$$I_k = \sum_{i\in S_k} \|\mathbf{w} \circ (\mathbf{x}_i - \mathbf{p}^{(k)})\|_2^2 = |S_k|\mathbf{w} \left(\mathbf{p}^{(k)} \circ (\mathbf{1} - \mathbf{p}^{(k)})\right), \tag{S2}$$

where '$\circ$' is the coefficient-wise product, '$\cdot$' is the Euclidean scalar product, and $\mathbf{1} = (1, \ldots, 1)$ as well as $\|\cdot\|_2$ denote Euclidean norm. Note that the variance of a cluster S_k having feature j is simply $p_j^{(k)}(1-p_j^{(k)})$. This means that the inertia I_k measures the heterogeneity of observations belonging to the same cluster, averaged over all features and weighted by some function $\mathbf{w}$. Here, we chose this weight function as $w_j = -\ln(p_j)$, with $p_j = 1M \sum_{i=1}^{M} X_{i,j}$. DIVCLUS-T minimizes the objective function

$$F = \sum_{k=1}^{K} I_k \quad . \tag{S3}$$

We refer to sets of observations from the last iteration of the algorithm by dropping the index k to simplify notation. For any given feature j, we can identify subsets $S_0^j = \{i \in S \mid X_{i,j} = 0\}$ and $S_1^j = \{i \in S \mid X_{i,j} = 1\}$ that do or do not contain feature j, respectively. The algorithm now identifies the feature

$$j_0 = \underset{j \in J}{argmin} \left(I_0^j + I_1^j \right), \tag{S4}$$

minimizing the inertias of S_1^j and S_0^j, respectively, together with the set

$$J = \left\{ 1 \leq j \leq N \,\middle|\, v_j = 1 \wedge min(|S_0^j|, |S_1^j|) > \theta \right\}. \tag{S5}$$

This way, a parent node k is split into two new leaf nodes containing $S_0^{j_0}$ and $S_1^{j_0}$, respectively. The split is made by separating observations having feature j_0 from those not having it. Vector $\mathbf{v}$ gives the features which are in principle allowed to perform the split operations which.

The algorithm stops once J is empty for each leaf node. Let L be the number of leaves and denote by F the sum of all leaf inertias. Now consider two leaf nodes with the same parent and remove those two leaves. You end up with a new tree of depths $L - 1$ and sum of inertias F'. This way one can identify the pair of leaves to remove such that $F' - F$ is minimal. Repeating this procedure $L - K$ times to arrive at a tree with K nodes representing sets $C_1, \ldots, C_K$ giving a partitioning of $\mathbf{X}$ into K disjoint clusters. Reasonable cut-off values for the number of clusters can be identified using the elbow method for the mean cluster inertia.

2 SUPPLEMENTARY FIGURES

3 SUPPLEMENTARY TABLES

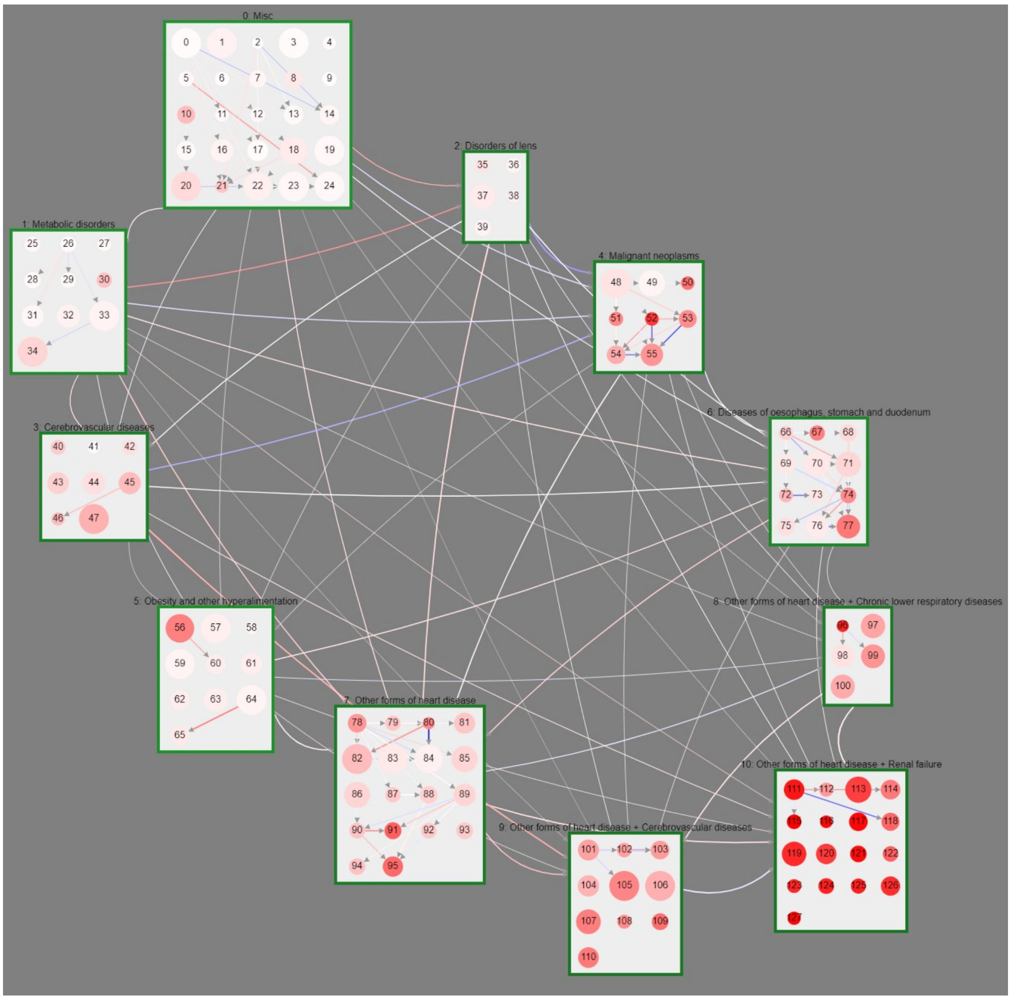

Figure S1: Controlled trajectory comparison of male and female patients of the matched control group. Here, link weights are given by the age-averaged sex risk difference SRD computed in the control group, instead of the DM group as in the main text. Blue (red) links indicate that the corresponding cluster transition is more (less) frequently observed in trajectories of male patients compared to female patients in the matched control group.

3.1 Macro-cluster condition tables

Malignant neoplasms (C00-C97)	✗
Obesity and other hyperalimentation (E65-E68)	✗
Metabolic disorders (E70-E90)	✗
Disorders of lens (H25-H28)	✗
Other forms of heart disease (I30-I52)	✗
Cerebrovascular diseases (I60-I69)	✗
Diseases of oesophagus, stomach and duodenum (K20-K31)	✗

Table S1: Inclusion and exclusion criteria for macro cluster 0. Female ratio: 42%, mean age of patients: 49.

Malignant neoplasms (C00-C97)	✗
Obesity and other hyperalimentation (E65-E68)	✗
Metabolic disorders (E70-E90)	✓
Disorders of lens (H25-H28)	✗
Other forms of heart disease (I30-I52)	✗
Cerebrovascular diseases (I60-I69)	✗
Diseases of oesophagus, stomach and duodenum (K20-K31)	✗

Table S2: Inclusion and exclusion criteria for macro cluster 1. Female ratio: 32%, mean age of patients: 37.

Malignant neoplasms (C00-C97)	✗
Obesity and other hyperalimentation (E65-E68)	✗
Disorders of lens (H25-H28)	✓
Other forms of heart disease (I30-I52)	✗
Cerebrovascular diseases (I60-I69)	✗
Diseases of oesophagus, stomach and duodenum (K20-K31)	✗

Table S3: Inclusion and exclusion criteria for macro cluster 2. Female ratio: 49%, mean age of patients: 66.

Malignant neoplasms (C00-C97)	✗
Obesity and other hyperalimentation (E65-E68)	✗
Other forms of heart disease (I30-I52)	✗
Cerebrovascular diseases (I60-I69)	✓
Diseases of oesophagus, stomach and duodenum (K20-K31)	✗

Table S4: Inclusion and exclusion criteria for macro cluster 3. Female ratio: 48%, mean age of patients: 44.

Malignant neoplasms (C00-C97)	✓
Obesity and other hyperalimentation (E65-E68)	✗
Other forms of heart disease (I30-I52)	✗
Diseases of oesophagus, stomach and duodenum (K20-K31)	✗

Table S5: Inclusion and exclusion criteria for macro cluster 4. Female ratio: 40%, mean age of patients: 59.

Obesity and other hyperalimentation (E65-E68)	✓
Other forms of heart disease (I30-I52)	✗
Diseases of oesophagus, stomach and duodenum (K20-K31)	✗

Table S6: Inclusion and exclusion criteria for macro cluster 5. Female ratio: 36%, mean age of patients: 58.

Other forms of heart disease (I30-I52)	✗
Diseases of oesophagus, stomach and duodenum (K20-K31)	✓

Table S7: Inclusion and exclusion criteria for macro cluster 6. Female ratio: 43%, mean age of patients: 63.

Other forms of heart disease (I30-I52)	✓
Cerebrovascular diseases (I60-I69)	✗
Chronic lower respiratory diseases (J40-J47)	✗
Renal failure (N17-N19)	✗

Table S8: Inclusion and exclusion criteria for macro cluster 7. Female ratio: 36%, mean age of patients: 71.

Other forms of heart disease (I30-I52)	✓
Cerebrovascular diseases (I60-I69)	✗
Chronic lower respiratory diseases (J40-J47)	✓
Renal failure (N17-N19)	✗

Table S9: Inclusion and exclusion criteria for macro cluster 8. Female ratio: 100%, mean age of patients: 55.

Other forms of heart disease (I30-I52)	✓
Cerebrovascular diseases (I60-I69)	✓
Renal failure (N17-N19)	✗

Table S10: Inclusion and exclusion criteria for macro cluster 9. Female ratio: 44%, mean age of patients: 69.

Other forms of heart disease (I30-I52)	✓
Renal failure (N17-N19)	✓

Table S11: Inclusion and exclusion criteria for macro cluster 10. Female ratio: 41%, mean age of patients: 42.

3.2 Cluster condition tables

Intestinal infectious diseases (A00-A09)	✗
Malignant neoplasms (C00-C97)	✗
Disorders of thyroid gland (E00-E07)	✗
Other disorders of glucose regulation and pancreatic internal secretion (E15-E16)	✗
Obesity and other hyperalimentation (E65-E68)	✗
Metabolic disorders (E70-E90)	✗
Mental and behavioural disorders due to psychoactive substance use (F10-F19)	✗
Episodic and paroxysmal disorders (G40-G47)	✗
Disorders of lens (H25-H28)	✗
Disorders of choroid and retina (H30-H36)	✗
Hypertensive diseases (I10-I15)	✗
Ischaemic heart diseases (I20-I25)	✗
Other forms of heart disease (I30-I52)	✗
Cerebrovascular diseases (I60-I69)	✗
Diseases of arteries, arterioles and capillaries (I70-I79)	✗
Diseases of veins, lymphatic vessels and lymph nodes, not elsewhere classified (I80-I89)	✗
Influenza and pneumonia (J09-J18)	✗
Other diseases of upper respiratory tract (J30-J39)	✗
Diseases of oesophagus, stomach and duodenum (K20-K31)	✗
Other diseases of intestines (K55-K63)	✗
Disorders of gallbladder, biliary tract and pancreas (K80-K87)	✗
Infections of the skin and subcutaneous tissue (L00-L08)	✗
Arthropathies (M00-M25)	✗
Dorsopathies (M40-M54)	✗
Soft tissue disorders (M60-M79)	✗
Other diseases of urinary system (N30-N39)	✗
Diseases of male genital organs (N40-N51)	✗
Noninflammatory disorders of female genital tract (N80-N98)	✗

Table S12: Inclusion and exclusion criteria for cluster 0. Female ratio: 42%, mean age of patients: 49.

Intestinal infectious diseases (A00-A09)	✗
Malignant neoplasms (C00-C97)	✗
Disorders of thyroid gland (E00-E07)	✗
Other disorders of glucose regulation and pancreatic internal secretion (E15-E16)	✗
Obesity and other hyperalimentation (E65-E68)	✗
Metabolic disorders (E70-E90)	✗
Mental and behavioural disorders due to psychoactive substance use (F10-F19)	✗
Episodic and paroxysmal disorders (G40-G47)	✗
Disorders of lens (H25-H28)	✗
Disorders of choroid and retina (H30-H36)	✗
Hypertensive diseases (I10-I15)	✗
Ischaemic heart diseases (I20-I25)	✗
Other forms of heart disease (I30-I52)	✗
Cerebrovascular diseases (I60-I69)	✗
Diseases of arteries, arterioles and capillaries (I70-I79)	✗
Diseases of veins, lymphatic vessels and lymph nodes, not elsewhere classified (I80-I89)	✗
Influenza and pneumonia (J09-J18)	✗
Other diseases of upper respiratory tract (J30-J39)	✓
Diseases of oesophagus, stomach and duodenum (K20-K31)	✗
Other diseases of intestines (K55-K63)	✗
Disorders of gallbladder, biliary tract and pancreas (K80-K87)	✗
Infections of the skin and subcutaneous tissue (L00-L08)	✗
Arthropathies (M00-M25)	✗
Dorsopathies (M40-M54)	✗
Soft tissue disorders (M60-M79)	✗
Other diseases of urinary system (N30-N39)	✗
Diseases of male genital organs (N40-N51)	✗
Noninflammatory disorders of female genital tract (N80-N98)	✗

Table S13: Inclusion and exclusion criteria for cluster 1. Female ratio: 32%, mean age of patients: 37.

Intestinal infectious diseases (A00-A09)	✗
Malignant neoplasms (C00-C97)	✗
Disorders of thyroid gland (E00-E07)	✗
Other disorders of glucose regulation and pancreatic internal secretion (E15-E16)	✗
Obesity and other hyperalimentation (E65-E68)	✗
Metabolic disorders (E70-E90)	✗
Mental and behavioural disorders due to psychoactive substance use (F10-F19)	✗
Episodic and paroxysmal disorders (G40-G47)	✗
Disorders of lens (H25-H28)	✗
Disorders of choroid and retina (H30-H36)	✗
Hypertensive diseases (I10-I15)	✓
Ischaemic heart diseases (I20-I25)	✗
Other forms of heart disease (I30-I52)	✗
Cerebrovascular diseases (I60-I69)	✗
Diseases of arteries, arterioles and capillaries (I70-I79)	✗
Diseases of veins, lymphatic vessels and lymph nodes, not elsewhere classified (I80-I89)	✗
Influenza and pneumonia (J09-J18)	✗
Diseases of oesophagus, stomach and duodenum (K20-K31)	✗
Other diseases of intestines (K55-K63)	✗
Disorders of gallbladder, biliary tract and pancreas (K80-K87)	✗
Infections of the skin and subcutaneous tissue (L00-L08)	✗
Arthropathies (M00-M25)	✗
Dorsopathies (M40-M54)	✗
Soft tissue disorders (M60-M79)	✗
Other diseases of urinary system (N30-N39)	✗
Diseases of male genital organs (N40-N51)	✗
Noninflammatory disorders of female genital tract (N80-N98)	✗

Table S14: Inclusion and exclusion criteria for cluster 2. Female ratio: 49%, mean age of patients: 66.

Intestinal infectious diseases (A00-A09)	✓
Malignant neoplasms (C00-C97)	✗
Disorders of thyroid gland (E00-E07)	✗
Other disorders of glucose regulation and pancreatic internal secretion (E15-E16)	✗
Obesity and other hyperalimentation (E65-E68)	✗
Metabolic disorders (E70-E90)	✗
Mental and behavioural disorders due to psychoactive substance use (F10-F19)	✗
Episodic and paroxysmal disorders (G40-G47)	✗
Disorders of lens (H25-H28)	✗
Disorders of choroid and retina (H30-H36)	✗
Ischaemic heart diseases (I20-I25)	✗
Other forms of heart disease (I30-I52)	✗
Cerebrovascular diseases (I60-I69)	✗
Diseases of arteries, arterioles and capillaries (I70-I79)	✗
Diseases of veins, lymphatic vessels and lymph nodes, not elsewhere classified (I80-I89)	✗
Influenza and pneumonia (J09-J18)	✗
Diseases of oesophagus, stomach and duodenum (K20-K31)	✗
Other diseases of intestines (K55-K63)	✗
Disorders of gallbladder, biliary tract and pancreas (K80-K87)	✗
Infections of the skin and subcutaneous tissue (L00-L08)	✗
Arthropathies (M00-M25)	✗
Dorsopathies (M40-M54)	✗
Soft tissue disorders (M60-M79)	✗
Other diseases of urinary system (N30-N39)	✗
Diseases of male genital organs (N40-N51)	✗
Noninflammatory disorders of female genital tract (N80-N98)	✗

Table S15: Inclusion and exclusion criteria for cluster 3. Female ratio: 48%, mean age of patients: 44.

Malignant neoplasms (C00-C97)	✗
Disorders of thyroid gland (E00-E07)	✗
Other disorders of glucose regulation and pancreatic internal secretion (E15-E16)	✗
Obesity and other hyperalimentation (E65-E68)	✗
Metabolic disorders (E70-E90)	✗
Mental and behavioural disorders due to psychoactive substance use (F10-F19)	✗
Episodic and paroxysmal disorders (G40-G47)	✓
Disorders of lens (H25-H28)	✗
Disorders of choroid and retina (H30-H36)	✗
Ischaemic heart diseases (I20-I25)	✗
Other forms of heart disease (I30-I52)	✗
Cerebrovascular diseases (I60-I69)	✗
Diseases of arteries, arterioles and capillaries (I70-I79)	✗
Diseases of veins, lymphatic vessels and lymph nodes, not elsewhere classified (I80-I89)	✗
Influenza and pneumonia (J09-J18)	✗
Diseases of oesophagus, stomach and duodenum (K20-K31)	✗
Other diseases of intestines (K55-K63)	✗
Disorders of gallbladder, biliary tract and pancreas (K80-K87)	✗
Infections of the skin and subcutaneous tissue (L00-L08)	✗
Arthropathies (M00-M25)	✗
Dorsopathies (M40-M54)	✗
Soft tissue disorders (M60-M79)	✗
Other diseases of urinary system (N30-N39)	✗
Diseases of male genital organs (N40-N51)	✗
Noninflammatory disorders of female genital tract (N80-N98)	✗

Table S16: Inclusion and exclusion criteria for cluster 4. Female ratio: 40%, mean age of patients: 59.

Malignant neoplasms (C00-C97)	✗
Disorders of thyroid gland (E00-E07)	✗
Other disorders of glucose regulation and pancreatic internal secretion (E15-E16)	✗
Obesity and other hyperalimentation (E65-E68)	✗
Metabolic disorders (E70-E90)	✗
Mental and behavioural disorders due to psychoactive substance use (F10-F19)	✗
Disorders of lens (H25-H28)	✗
Disorders of choroid and retina (H30-H36)	✗
Ischaemic heart diseases (I20-I25)	✗
Other forms of heart disease (I30-I52)	✗
Cerebrovascular diseases (I60-I69)	✗
Diseases of arteries, arterioles and capillaries (I70-I79)	✗
Diseases of veins, lymphatic vessels and lymph nodes, not elsewhere classified (I80-I89)	✗
Influenza and pneumonia (J09-J18)	✗
Diseases of oesophagus, stomach and duodenum (K20-K31)	✗
Other diseases of intestines (K55-K63)	✗
Disorders of gallbladder, biliary tract and pancreas (K80-K87)	✗
Infections of the skin and subcutaneous tissue (L00-L08)	✗
Arthropathies (M00-M25)	✗
Dorsopathies (M40-M54)	✗
Soft tissue disorders (M60-M79)	✓
Other diseases of urinary system (N30-N39)	✗
Diseases of male genital organs (N40-N51)	✗
Noninflammatory disorders of female genital tract (N80-N98)	✗

Table S17: Inclusion and exclusion criteria for cluster 5. Female ratio: 36%, mean age of patients: 58.

Malignant neoplasms (C00-C97)	✗
Disorders of thyroid gland (E00-E07)	✗
Other disorders of glucose regulation and pancreatic internal secretion (E15-E16)	✗
Obesity and other hyperalimentation (E65-E68)	✗
Metabolic disorders (E70-E90)	✗
Mental and behavioural disorders due to psychoactive substance use (F10-F19)	✗
Disorders of lens (H25-H28)	✗
Disorders of choroid and retina (H30-H36)	✗
Ischaemic heart diseases (I20-I25)	✗
Other forms of heart disease (I30-I52)	✗
Cerebrovascular diseases (I60-I69)	✗
Diseases of arteries, arterioles and capillaries (I70-I79)	✗
Diseases of veins, lymphatic vessels and lymph nodes, not elsewhere classified (I80-I89)	✗
Influenza and pneumonia (J09-J18)	✗
Diseases of oesophagus, stomach and duodenum (K20-K31)	✗
Other diseases of intestines (K55-K63)	✓
Disorders of gallbladder, biliary tract and pancreas (K80-K87)	✗
Infections of the skin and subcutaneous tissue (L00-L08)	✗
Arthropathies (M00-M25)	✗
Dorsopathies (M40-M54)	✗
Other diseases of urinary system (N30-N39)	✗
Diseases of male genital organs (N40-N51)	✗
Noninflammatory disorders of female genital tract (N80-N98)	✗

Table S18: Inclusion and exclusion criteria for cluster 6. Female ratio: 43%, mean age of patients: 63.

Malignant neoplasms (C00-C97)	✗
Disorders of thyroid gland (E00-E07)	✗
Other disorders of glucose regulation and pancreatic internal secretion (E15-E16)	✗
Obesity and other hyperalimentation (E65-E68)	✗
Metabolic disorders (E70-E90)	✗
Mental and behavioural disorders due to psychoactive substance use (F10-F19)	✗
Disorders of lens (H25-H28)	✗
Disorders of choroid and retina (H30-H36)	✗
Ischaemic heart diseases (I20-I25)	✗
Other forms of heart disease (I30-I52)	✗
Cerebrovascular diseases (I60-I69)	✗
Diseases of arteries, arterioles and capillaries (I70-I79)	✓
Diseases of veins, lymphatic vessels and lymph nodes, not elsewhere classified (I80-I89)	✗
Influenza and pneumonia (J09-J18)	✗
Diseases of oesophagus, stomach and duodenum (K20-K31)	✗
Disorders of gallbladder, biliary tract and pancreas (K80-K87)	✗
Infections of the skin and subcutaneous tissue (L00-L08)	✗
Arthropathies (M00-M25)	✗
Dorsopathies (M40-M54)	✗
Other diseases of urinary system (N30-N39)	✗
Diseases of male genital organs (N40-N51)	✗
Noninflammatory disorders of female genital tract (N80-N98)	✗

Table S19: Inclusion and exclusion criteria for cluster 7. Female ratio: 36%, mean age of patients: 71.

Malignant neoplasms (C00-C97)	✗
Disorders of thyroid gland (E00-E07)	✗
Other disorders of glucose regulation and pancreatic internal secretion (E15-E16)	✗
Obesity and other hyperalimentation (E65-E68)	✗
Metabolic disorders (E70-E90)	✗
Mental and behavioural disorders due to psychoactive substance use (F10-F19)	✗
Disorders of lens (H25-H28)	✗
Disorders of choroid and retina (H30-H36)	✗
Ischaemic heart diseases (I20-I25)	✗
Other forms of heart disease (I30-I52)	✗
Cerebrovascular diseases (I60-I69)	✗
Diseases of veins, lymphatic vessels and lymph nodes, not elsewhere classified (I80-I89)	✗
Influenza and pneumonia (J09-J18)	✗
Diseases of oesophagus, stomach and duodenum (K20-K31)	✗
Disorders of gallbladder, biliary tract and pancreas (K80-K87)	✗
Infections of the skin and subcutaneous tissue (L00-L08)	✗
Arthropathies (M00-M25)	✗
Dorsopathies (M40-M54)	✗
Other diseases of urinary system (N30-N39)	✗
Diseases of male genital organs (N40-N51)	✗
Noninflammatory disorders of female genital tract (N80-N98)	✓

Table S20: Inclusion and exclusion criteria for cluster 8. Female ratio: 100%, mean age of patients: 55.

Malignant neoplasms (C00-C97)	✗
Disorders of thyroid gland (E00-E07)	✗
Other disorders of glucose regulation and pancreatic internal secretion (E15-E16)	✗
Obesity and other hyperalimentation (E65-E68)	✗
Metabolic disorders (E70-E90)	✗
Mental and behavioural disorders due to psychoactive substance use (F10-F19)	✗
Disorders of lens (H25-H28)	✗
Disorders of choroid and retina (H30-H36)	✗
Ischaemic heart diseases (I20-I25)	✗
Other forms of heart disease (I30-I52)	✗
Cerebrovascular diseases (I60-I69)	✗
Diseases of veins, lymphatic vessels and lymph nodes, not elsewhere classified (I80-I89)	✗
Influenza and pneumonia (J09-J18)	✓
Diseases of oesophagus, stomach and duodenum (K20-K31)	✗
Disorders of gallbladder, biliary tract and pancreas (K80-K87)	✗
Infections of the skin and subcutaneous tissue (L00-L08)	✗
Arthropathies (M00-M25)	✗
Dorsopathies (M40-M54)	✗
Other diseases of urinary system (N30-N39)	✗
Diseases of male genital organs (N40-N51)	✗

Table S21: Inclusion and exclusion criteria for cluster 9. Female ratio: 44%, mean age of patients: 69.

Malignant neoplasms (C00-C97)	✗
Disorders of thyroid gland (E00-E07)	✗
Other disorders of glucose regulation and pancreatic internal secretion (E15-E16)	✓
Obesity and other hyperalimentation (E65-E68)	✗
Metabolic disorders (E70-E90)	✗
Mental and behavioural disorders due to psychoactive substance use (F10-F19)	✗
Disorders of lens (H25-H28)	✗
Disorders of choroid and retina (H30-H36)	✗
Ischaemic heart diseases (I20-I25)	✗
Other forms of heart disease (I30-I52)	✗
Cerebrovascular diseases (I60-I69)	✗
Diseases of veins, lymphatic vessels and lymph nodes, not elsewhere classified (I80-I89)	✗
Diseases of oesophagus, stomach and duodenum (K20-K31)	✗
Disorders of gallbladder, biliary tract and pancreas (K80-K87)	✗
Infections of the skin and subcutaneous tissue (L00-L08)	✗
Arthropathies (M00-M25)	✗
Dorsopathies (M40-M54)	✗
Other diseases of urinary system (N30-N39)	✗
Diseases of male genital organs (N40-N51)	✗

Table S22: Inclusion and exclusion criteria for cluster 10. Female ratio: 41%, mean age of patients: 42.

Malignant neoplasms (C00-C97)	✗
Disorders of thyroid gland (E00-E07)	✗
Obesity and other hyperalimentation (E65-E68)	✗
Metabolic disorders (E70-E90)	✗
Mental and behavioural disorders due to psychoactive substance use (F10-F19)	✗
Disorders of lens (H25-H28)	✗
Disorders of choroid and retina (H30-H36)	✗
Ischaemic heart diseases (I20-I25)	✗
Other forms of heart disease (I30-I52)	✗
Cerebrovascular diseases (I60-I69)	✗
Diseases of veins, lymphatic vessels and lymph nodes, not elsewhere classified (I80-I89)	✗
Diseases of oesophagus, stomach and duodenum (K20-K31)	✗
Disorders of gallbladder, biliary tract and pancreas (K80-K87)	✗
Infections of the skin and subcutaneous tissue (L00-L08)	✓
Arthropathies (M00-M25)	✗
Dorsopathies (M40-M54)	✗
Other diseases of urinary system (N30-N39)	✗
Diseases of male genital organs (N40-N51)	✗

Table S23: Inclusion and exclusion criteria for cluster 11. Female ratio: 35%, mean age of patients: 54.

Malignant neoplasms (C00-C97)	✗
Disorders of thyroid gland (E00-E07)	✓
Obesity and other hyperalimentation (E65-E68)	✗
Metabolic disorders (E70-E90)	✗
Mental and behavioural disorders due to psychoactive substance use (F10-F19)	✗
Disorders of lens (H25-H28)	✗
Disorders of choroid and retina (H30-H36)	✗
Ischaemic heart diseases (I20-I25)	✗
Other forms of heart disease (I30-I52)	✗
Cerebrovascular diseases (I60-I69)	✗
Diseases of veins, lymphatic vessels and lymph nodes, not elsewhere classified (I80-I89)	✗
Diseases of oesophagus, stomach and duodenum (K20-K31)	✗
Disorders of gallbladder, biliary tract and pancreas (K80-K87)	✗
Arthropathies (M00-M25)	✗
Dorsopathies (M40-M54)	✗
Other diseases of urinary system (N30-N39)	✗
Diseases of male genital organs (N40-N51)	✗

Table S24: Inclusion and exclusion criteria for cluster 12. Female ratio: 68%, mean age of patients: 57.

Malignant neoplasms (C00-C97)	✗
Obesity and other hyperalimentation (E65-E68)	✗
Metabolic disorders (E70-E90)	✗
Mental and behavioural disorders due to psychoactive substance use (F10-F19)	✗
Disorders of lens (H25-H28)	✗
Disorders of choroid and retina (H30-H36)	✗
Ischaemic heart diseases (I20-I25)	✗
Other forms of heart disease (I30-I52)	✗
Cerebrovascular diseases (I60-I69)	✗
Diseases of veins, lymphatic vessels and lymph nodes, not elsewhere classified (I80-I89)	✓
Diseases of oesophagus, stomach and duodenum (K20-K31)	✗
Disorders of gallbladder, biliary tract and pancreas (K80-K87)	✗
Arthropathies (M00-M25)	✗
Dorsopathies (M40-M54)	✗
Other diseases of urinary system (N30-N39)	✗
Diseases of male genital organs (N40-N51)	✗

Table S25: Inclusion and exclusion criteria for cluster 13. Female ratio: 50%, mean age of patients: 63.

Malignant neoplasms (C00-C97)	✗
Obesity and other hyperalimentation (E65-E68)	✗
Metabolic disorders (E70-E90)	✗
Mental and behavioural disorders due to psychoactive substance use (F10-F19)	✗
Disorders of lens (H25-H28)	✗
Disorders of choroid and retina (H30-H36)	✗
Ischaemic heart diseases (I20-I25)	✗
Other forms of heart disease (I30-I52)	✗
Cerebrovascular diseases (I60-I69)	✗
Diseases of oesophagus, stomach and duodenum (K20-K31)	✗
Disorders of gallbladder, biliary tract and pancreas (K80-K87)	✗
Arthropathies (M00-M25)	✗
Dorsopathies (M40-M54)	✗
Other diseases of urinary system (N30-N39)	✗
Diseases of male genital organs (N40-N51)	✓

Table S26: Inclusion and exclusion criteria for cluster 14. Female ratio: 0%, mean age of patients: 58.

Malignant neoplasms (C00-C97)	✗
Obesity and other hyperalimentation (E65-E68)	✗
Metabolic disorders (E70-E90)	✗
Mental and behavioural disorders due to psychoactive substance use (F10-F19)	✗
Disorders of lens (H25-H28)	✗
Disorders of choroid and retina (H30-H36)	✗
Ischaemic heart diseases (I20-I25)	✗
Other forms of heart disease (I30-I52)	✗
Cerebrovascular diseases (I60-I69)	✗
Diseases of oesophagus, stomach and duodenum (K20-K31)	✗
Disorders of gallbladder, biliary tract and pancreas (K80-K87)	✓
Arthropathies (M00-M25)	✗
Dorsopathies (M40-M54)	✗
Other diseases of urinary system (N30-N39)	✗

Table S27: Inclusion and exclusion criteria for cluster 15. Female ratio: 49%, mean age of patients: 65.

Malignant neoplasms (C00-C97)	✗
Obesity and other hyperalimentation (E65-E68)	✗
Metabolic disorders (E70-E90)	✗
Mental and behavioural disorders due to psychoactive substance use (F10-F19)	✗
Disorders of lens (H25-H28)	✗
Disorders of choroid and retina (H30-H36)	✓
Ischaemic heart diseases (I20-I25)	✗
Other forms of heart disease (I30-I52)	✗
Cerebrovascular diseases (I60-I69)	✗
Diseases of oesophagus, stomach and duodenum (K20-K31)	✗
Arthropathies (M00-M25)	✗
Dorsopathies (M40-M54)	✗
Other diseases of urinary system (N30-N39)	✗

Table S28: Inclusion and exclusion criteria for cluster 16. Female ratio: 39%, mean age of patients: 60.

Malignant neoplasms (C00-C97)	✗
Obesity and other hyperalimentation (E65-E68)	✗
Metabolic disorders (E70-E90)	✗
Mental and behavioural disorders due to psychoactive substance use (F10-F19)	✗
Disorders of lens (H25-H28)	✗
Ischaemic heart diseases (I20-I25)	✓
Other forms of heart disease (I30-I52)	✗
Cerebrovascular diseases (I60-I69)	✗
Diseases of oesophagus, stomach and duodenum (K20-K31)	✗
Arthropathies (M00-M25)	✗
Dorsopathies (M40-M54)	✗
Other diseases of urinary system (N30-N39)	✗

Table S29: Inclusion and exclusion criteria for cluster 17. Female ratio: 37%, mean age of patients: 71.

Malignant neoplasms (C00-C97)	✗
Obesity and other hyperalimentation (E65-E68)	✗
Metabolic disorders (E70-E90)	✗
Organic, including symptomatic, mental disorders (F00-F09)	✗
Mental and behavioural disorders due to psychoactive substance use (F10-F19)	✗
Disorders of lens (H25-H28)	✗
Other forms of heart disease (I30-I52)	✗
Cerebrovascular diseases (I60-I69)	✗
Diseases of oesophagus, stomach and duodenum (K20-K31)	✗
Arthropathies (M00-M25)	✗
Dorsopathies (M40-M54)	✗
Other diseases of urinary system (N30-N39)	✓

Table S30: Inclusion and exclusion criteria for cluster 18. Female ratio: 67%, mean age of patients: 69.

Malignant neoplasms (C00-C97)	✗
Obesity and other hyperalimentation (E65-E68)	✗
Metabolic disorders (E70-E90)	✗
Organic, including symptomatic, mental disorders (F00-F09)	✓
Mental and behavioural disorders due to psychoactive substance use (F10-F19)	✗
Disorders of lens (H25-H28)	✗
Other forms of heart disease (I30-I52)	✗
Cerebrovascular diseases (I60-I69)	✗
Diseases of oesophagus, stomach and duodenum (K20-K31)	✗
Arthropathies (M00-M25)	✗
Dorsopathies (M40-M54)	✗
Other diseases of urinary system (N30-N39)	✓

Table S31: Inclusion and exclusion criteria for cluster 19. Female ratio: 74%, mean age of patients: 85.

Malignant neoplasms (C00-C97)	✗
Obesity and other hyperalimentation (E65-E68)	✗
Metabolic disorders (E70-E90)	✗
Mental and behavioural disorders due to psychoactive substance use (F10-F19)	✓
Disorders of lens (H25-H28)	✗
Other forms of heart disease (I30-I52)	✗
Cerebrovascular diseases (I60-I69)	✗
Diseases of oesophagus, stomach and duodenum (K20-K31)	✗
Arthropathies (M00-M25)	✗
Dorsopathies (M40-M54)	✗

Table S32: Inclusion and exclusion criteria for cluster 20. Female ratio: 23%, mean age of patients: 55.

Malignant neoplasms (C00-C97)	✗
Obesity and other hyperalimentation (E65-E68)	✗
Metabolic disorders (E70-E90)	✗
Disorders of lens (H25-H28)	✗
Other forms of heart disease (I30-I52)	✗
Cerebrovascular diseases (I60-I69)	✗
Diseases of oesophagus, stomach and duodenum (K20-K31)	✗
Arthropathies (M00-M25)	✓
Dorsopathies (M40-M54)	✗
Soft tissue disorders (M60-M79)	✗

Table S33: Inclusion and exclusion criteria for cluster 21. Female ratio: 51%, mean age of patients: 67.

Malignant neoplasms (C00-C97)	✗
Obesity and other hyperalimentation (E65-E68)	✗
Metabolic disorders (E70-E90)	✗
Disorders of lens (H25-H28)	✗
Other forms of heart disease (I30-I52)	✗
Cerebrovascular diseases (I60-I69)	✗
Diseases of oesophagus, stomach and duodenum (K20-K31)	✗
Arthropathies (M00-M25)	✗
Dorsopathies (M40-M54)	✓

Table S34: Inclusion and exclusion criteria for cluster 22. Female ratio: 49%, mean age of patients: 64.

Malignant neoplasms (C00-C97)	✗
Obesity and other hyperalimentation (E65-E68)	✗
Metabolic disorders (E70-E90)	✗
Disorders of lens (H25-H28)	✗
Other forms of heart disease (I30-I52)	✗
Cerebrovascular diseases (I60-I69)	✗
Diseases of oesophagus, stomach and duodenum (K20-K31)	✗
Arthropathies (M00-M25)	✓
Dorsopathies (M40-M54)	✓
Soft tissue disorders (M60-M79)	✗

Table S35: Inclusion and exclusion criteria for cluster 23. Female ratio: 59%, mean age of patients: 70.

Malignant neoplasms (C00-C97)	✗
Obesity and other hyperalimentation (E65-E68)	✗
Metabolic disorders (E70-E90)	✗
Disorders of lens (H25-H28)	✗
Other forms of heart disease (I30-I52)	✗
Cerebrovascular diseases (I60-I69)	✗
Diseases of oesophagus, stomach and duodenum (K20-K31)	✗
Arthropathies (M00-M25)	✓
Soft tissue disorders (M60-M79)	✓

Table S36: Inclusion and exclusion criteria for cluster 24. Female ratio: 43%, mean age of patients: 63.

Malignant neoplasms (C00-C97)	✗
Obesity and other hyperalimentation (E65-E68)	✗
Metabolic disorders (E70-E90)	✗
Organic, including symptomatic, mental disorders (F00-F09)	✗
Episodic and paroxysmal disorders (G40-G47)	✗
Disorders of lens (H25-H28)	✗
Other forms of heart disease (I30-I52)	✗
Cerebrovascular diseases (I60-I69)	✓
Diseases of arteries, arterioles and capillaries (I70-I79)	✗
Diseases of oesophagus, stomach and duodenum (K20-K31)	✗
Other diseases of urinary system (N30-N39)	✗

Table S37: Inclusion and exclusion criteria for cluster 25. Female ratio: 43%, mean age of patients: 74.

Malignant neoplasms (C00-C97)	✗
Disorders of thyroid gland (E00-E07)	✗
Obesity and other hyperalimentation (E65-E68)	✗
Metabolic disorders (E70-E90)	✓
Organic, including symptomatic, mental disorders (F00-F09)	✗
Mental and behavioural disorders due to psychoactive substance use (F10-F19)	✗
Disorders of lens (H25-H28)	✗
Ischaemic heart diseases (I20-I25)	✗
Other forms of heart disease (I30-I52)	✗
Cerebrovascular diseases (I60-I69)	✗
Diseases of oesophagus, stomach and duodenum (K20-K31)	✗
Diseases of liver (K70-K77)	✗
Arthropathies (M00-M25)	✗
Dorsopathies (M40-M54)	✗

Table S38: Inclusion and exclusion criteria for cluster 26. Female ratio: 43%, mean age of patients: 60.

Malignant neoplasms (C00-C97)	✗
Disorders of thyroid gland (E00-E07)	✓
Obesity and other hyperalimentation (E65-E68)	✗
Metabolic disorders (E70-E90)	✓
Organic, including symptomatic, mental disorders (F00-F09)	✗
Mental and behavioural disorders due to psychoactive substance use (F10-F19)	✗
Disorders of lens (H25-H28)	✗
Ischaemic heart diseases (I20-I25)	✗
Other forms of heart disease (I30-I52)	✗
Cerebrovascular diseases (I60-I69)	✗
Diseases of oesophagus, stomach and duodenum (K20-K31)	✗
Diseases of liver (K70-K77)	✗
Arthropathies (M00-M25)	✗
Dorsopathies (M40-M54)	✗

Table S39: Inclusion and exclusion criteria for cluster 27. Female ratio: 68%, mean age of patients: 63.

Malignant neoplasms (C00-C97)	✗
Obesity and other hyperalimentation (E65-E68)	✗
Metabolic disorders (E70-E90)	✓
Organic, including symptomatic, mental disorders (F00-F09)	✗
Mental and behavioural disorders due to psychoactive substance use (F10-F19)	✗
Disorders of lens (H25-H28)	✗
Ischaemic heart diseases (I20-I25)	✗
Other forms of heart disease (I30-I52)	✗
Cerebrovascular diseases (I60-I69)	✗
Diseases of oesophagus, stomach and duodenum (K20-K31)	✗
Diseases of liver (K70-K77)	✗
Arthropathies (M00-M25)	✓
Dorsopathies (M40-M54)	✗

Table S40: Inclusion and exclusion criteria for cluster 28. Female ratio: 50%, mean age of patients: 66.

Malignant neoplasms (C00-C97)	✗
Obesity and other hyperalimentation (E65-E68)	✗
Metabolic disorders (E70-E90)	✓
Organic, including symptomatic, mental disorders (F00-F09)	✗
Mental and behavioural disorders due to psychoactive substance use (F10-F19)	✓
Disorders of lens (H25-H28)	✗
Ischaemic heart diseases (I20-I25)	✗
Other forms of heart disease (I30-I52)	✗
Cerebrovascular diseases (I60-I69)	✗
Diseases of oesophagus, stomach and duodenum (K20-K31)	✗
Diseases of liver (K70-K77)	✗
Dorsopathies (M40-M54)	✗

Table S41: Inclusion and exclusion criteria for cluster 29. Female ratio: 24%, mean age of patients: 54.

Malignant neoplasms (C00-C97)	✗
Obesity and other hyperalimentation (E65-E68)	✗
Metabolic disorders (E70-E90)	✓
Organic, including symptomatic, mental disorders (F00-F09)	✓
Disorders of lens (H25-H28)	✗
Ischaemic heart diseases (I20-I25)	✗
Other forms of heart disease (I30-I52)	✗
Cerebrovascular diseases (I60-I69)	✗
Diseases of oesophagus, stomach and duodenum (K20-K31)	✗
Diseases of liver (K70-K77)	✗
Dorsopathies (M40-M54)	✗

Table S42: Inclusion and exclusion criteria for cluster 30. Female ratio: 70%, mean age of patients: 83.

Malignant neoplasms (C00-C97)	✗
Obesity and other hyperalimentation (E65-E68)	✗
Metabolic disorders (E70-E90)	✓
Disorders of lens (H25-H28)	✗
Ischaemic heart diseases (I20-I25)	✗
Other forms of heart disease (I30-I52)	✗
Cerebrovascular diseases (I60-I69)	✗
Diseases of oesophagus, stomach and duodenum (K20-K31)	✗
Diseases of liver (K70-K77)	✗
Dorsopathies (M40-M54)	✓

Table S43: Inclusion and exclusion criteria for cluster 31. Female ratio: 50%, mean age of patients: 64.

Malignant neoplasms (C00-C97)	✗
Obesity and other hyperalimentation (E65-E68)	✗
Metabolic disorders (E70-E90)	✓
Disorders of lens (H25-H28)	✗
Ischaemic heart diseases (I20-I25)	✗
Other forms of heart disease (I30-I52)	✗
Cerebrovascular diseases (I60-I69)	✗
Diseases of oesophagus, stomach and duodenum (K20-K31)	✗
Diseases of liver (K70-K77)	✓

Table S44: Inclusion and exclusion criteria for cluster 32. Female ratio: 36%, mean age of patients: 59.

Malignant neoplasms (C00-C97)	✗
Obesity and other hyperalimentation (E65-E68)	✗
Metabolic disorders (E70-E90)	✓
Mental and behavioural disorders due to psychoactive substance use (F10-F19)	✗
Disorders of lens (H25-H28)	✗
Ischaemic heart diseases (I20-I25)	✓
Other forms of heart disease (I30-I52)	✗
Cerebrovascular diseases (I60-I69)	✗
Diseases of arteries, arterioles and capillaries (I70-I79)	✗
Diseases of oesophagus, stomach and duodenum (K20-K31)	✗

Table S45: Inclusion and exclusion criteria for cluster 33. Female ratio: 35%, mean age of patients: 68.

Malignant neoplasms (C00-C97)	✗
Obesity and other hyperalimentation (E65-E68)	✗
Metabolic disorders (E70-E90)	✓
Mental and behavioural disorders due to psychoactive substance use (F10-F19)	✗
Disorders of lens (H25-H28)	✗
Ischaemic heart diseases (I20-I25)	✓
Other forms of heart disease (I30-I52)	✗
Cerebrovascular diseases (I60-I69)	✗
Diseases of arteries, arterioles and capillaries (I70-I79)	✓
Diseases of oesophagus, stomach and duodenum (K20-K31)	✗

Table S46: Inclusion and exclusion criteria for cluster 34. Female ratio: 34%, mean age of patients: 71.

Malignant neoplasms (C00-C97)	✗
Obesity and other hyperalimentation (E65-E68)	✗
Metabolic disorders (E70-E90)	✓
Mental and behavioural disorders due to psychoactive substance use (F10-F19)	✓
Disorders of lens (H25-H28)	✗
Ischaemic heart diseases (I20-I25)	✓
Other forms of heart disease (I30-I52)	✗
Cerebrovascular diseases (I60-I69)	✗
Diseases of oesophagus, stomach and duodenum (K20-K31)	✗

Table S47: Inclusion and exclusion criteria for cluster 35. Female ratio: 18%, mean age of patients: 60.

Malignant neoplasms (C00-C97)	✗
Obesity and other hyperalimentation (E65-E68)	✗
Metabolic disorders (E70-E90)	✓
Organic, including symptomatic, mental disorders (F00-F09)	✗
Episodic and paroxysmal disorders (G40-G47)	✗
Disorders of lens (H25-H28)	✗
Other forms of heart disease (I30-I52)	✗
Cerebrovascular diseases (I60-I69)	✓
Diseases of arteries, arterioles and capillaries (I70-I79)	✗
Diseases of oesophagus, stomach and duodenum (K20-K31)	✗
Other diseases of urinary system (N30-N39)	✗

Table S48: Inclusion and exclusion criteria for cluster 36. Female ratio: 37%, mean age of patients: 70.

Malignant neoplasms (C00-C97)	✗
Obesity and other hyperalimentation (E65-E68)	✗
Organic, including symptomatic, mental disorders (F00-F09)	✗
Episodic and paroxysmal disorders (G40-G47)	✗
Disorders of lens (H25-H28)	✗
Other forms of heart disease (I30-I52)	✗
Cerebrovascular diseases (I60-I69)	✓
Diseases of arteries, arterioles and capillaries (I70-I79)	✗
Diseases of oesophagus, stomach and duodenum (K20-K31)	✗
Other diseases of urinary system (N30-N39)	✓

Table S49: Inclusion and exclusion criteria for cluster 37. Female ratio: 68%, mean age of patients: 78.

Malignant neoplasms (C00-C97)	✗
Obesity and other hyperalimentation (E65-E68)	✗
Disorders of lens (H25-H28)	✓
Disorders of choroid and retina (H30-H36)	✗
Glaucoma (H40-H42)	✗
Other forms of heart disease (I30-I52)	✗
Cerebrovascular diseases (I60-I69)	✗
Diseases of oesophagus, stomach and duodenum (K20-K31)	✗
Arthropathies (M00-M25)	✗

Table S50: Inclusion and exclusion criteria for cluster 38. Female ratio: 54%, mean age of patients: 74.

Malignant neoplasms (C00-C97)	✗
Obesity and other hyperalimentation (E65-E68)	✗
Disorders of lens (H25-H28)	✓
Disorders of choroid and retina (H30-H36)	✗
Glaucoma (H40-H42)	✓
Other forms of heart disease (I30-I52)	✗
Cerebrovascular diseases (I60-I69)	✗
Diseases of oesophagus, stomach and duodenum (K20-K31)	✗
Arthropathies (M00-M25)	✗

Table S51: Inclusion and exclusion criteria for cluster 39. Female ratio: 59%, mean age of patients: 76.

Malignant neoplasms (C00-C97)	✗
Obesity and other hyperalimentation (E65-E68)	✗
Disorders of lens (H25-H28)	✓
Disorders of choroid and retina (H30-H36)	✗
Other forms of heart disease (I30-I52)	✗
Cerebrovascular diseases (I60-I69)	✗
Diseases of oesophagus, stomach and duodenum (K20-K31)	✗
Arthropathies (M00-M25)	✓

Table S52: Inclusion and exclusion criteria for cluster 40. Female ratio: 65%, mean age of patients: 76.

Malignant neoplasms (C00-C97)	✗
Obesity and other hyperalimentation (E65-E68)	✗
Disorders of lens (H25-H28)	✓
Disorders of choroid and retina (H30-H36)	✓
Disorders of vitreous body and globe (H43-H45)	✗
Other forms of heart disease (I30-I52)	✗
Cerebrovascular diseases (I60-I69)	✗
Diseases of oesophagus, stomach and duodenum (K20-K31)	✗

Table S53: Inclusion and exclusion criteria for cluster 41. Female ratio: 56%, mean age of patients: 73.

Malignant neoplasms (C00-C97)	✗
Obesity and other hyperalimentation (E65-E68)	✗
Disorders of lens (H25-H28)	✓
Disorders of choroid and retina (H30-H36)	✓
Disorders of vitreous body and globe (H43-H45)	✓
Other forms of heart disease (I30-I52)	✗
Cerebrovascular diseases (I60-I69)	✗
Diseases of oesophagus, stomach and duodenum (K20-K31)	✗

Table S54: Inclusion and exclusion criteria for cluster 42. Female ratio: 42%, mean age of patients: 67.

Malignant neoplasms (C00-C97)	✗
Obesity and other hyperalimentation (E65-E68)	✗
Organic, including symptomatic, mental disorders (F00-F09)	✗
Episodic and paroxysmal disorders (G40-G47)	✗
Disorders of lens (H25-H28)	✓
Other forms of heart disease (I30-I52)	✗
Cerebrovascular diseases (I60-I69)	✓
Diseases of arteries, arterioles and capillaries (I70-I79)	✗
Diseases of oesophagus, stomach and duodenum (K20-K31)	✗

Table S55: Inclusion and exclusion criteria for cluster 43. Female ratio: 55%, mean age of patients: 78.

Malignant neoplasms (C00-C97)	✗
Obesity and other hyperalimentation (E65-E68)	✗
Organic, including symptomatic, mental disorders (F00-F09)	✗
Episodic and paroxysmal disorders (G40-G47)	✗
Other forms of heart disease (I30-I52)	✗
Cerebrovascular diseases (I60-I69)	✓
Diseases of arteries, arterioles and capillaries (I70-I79)	✓
Diseases of oesophagus, stomach and duodenum (K20-K31)	✗

Table S56: Inclusion and exclusion criteria for cluster 44. Female ratio: 38%, mean age of patients: 73.

Malignant neoplasms (C00-C97)	✗
Obesity and other hyperalimentation (E65-E68)	✗
Organic, including symptomatic, mental disorders (F00-F09)	✗
Episodic and paroxysmal disorders (G40-G47)	✓
Other forms of heart disease (I30-I52)	✗
Cerebrovascular diseases (I60-I69)	✓
Diseases of oesophagus, stomach and duodenum (K20-K31)	✗

Table S57: Inclusion and exclusion criteria for cluster 45. Female ratio: 40%, mean age of patients: 72.

Malignant neoplasms (C00-C97)	✗
Obesity and other hyperalimentation (E65-E68)	✗
Organic, including symptomatic, mental disorders (F00-F09)	✓
Other degenerative diseases of the nervous system (G30-G32)	✗
Other forms of heart disease (I30-I52)	✗
Cerebrovascular diseases (I60-I69)	✓
Diseases of oesophagus, stomach and duodenum (K20-K31)	✗

Table S58: Inclusion and exclusion criteria for cluster 46. Female ratio: 58%, mean age of patients: 81.

Malignant neoplasms (C00-C97)	✗
Obesity and other hyperalimentation (E65-E68)	✗
Organic, including symptomatic, mental disorders (F00-F09)	✓
Other degenerative diseases of the nervous system (G30-G32)	✓
Other forms of heart disease (I30-I52)	✗
Cerebrovascular diseases (I60-I69)	✓
Diseases of oesophagus, stomach and duodenum (K20-K31)	✗

Table S59: Inclusion and exclusion criteria for cluster 47. Female ratio: 64%, mean age of patients: 85.

Malignant neoplasms (C00-C97)	✓
Neoplasms of uncertain or unknown behaviour (D37-D48)	✗
Aplastic and other anaemias (D60-D64)	✗
Obesity and other hyperalimentation (E65-E68)	✗
Disorders of lens (H25-H28)	✗
Other forms of heart disease (I30-I52)	✗
Chronic lower respiratory diseases (J40-J47)	✗
Diseases of oesophagus, stomach and duodenum (K20-K31)	✗
Other diseases of intestines (K55-K63)	✗
Disorders of gallbladder, biliary tract and pancreas (K80-K87)	✗
Other diseases of urinary system (N30-N39)	✗

Table S60: Inclusion and exclusion criteria for cluster 48. Female ratio: 40%, mean age of patients: 70.

Malignant neoplasms (C00-C97)	✓
Neoplasms of uncertain or unknown behaviour (D37-D48)	✗
Aplastic and other anaemias (D60-D64)	✗
Obesity and other hyperalimentation (E65-E68)	✗
Disorders of lens (H25-H28)	✗
Other forms of heart disease (I30-I52)	✗
Chronic lower respiratory diseases (J40-J47)	✓
Diseases of oesophagus, stomach and duodenum (K20-K31)	✗
Other diseases of intestines (K55-K63)	✗
Disorders of gallbladder, biliary tract and pancreas (K80-K87)	✗
Other diseases of urinary system (N30-N39)	✗

Table S61: Inclusion and exclusion criteria for cluster 49. Female ratio: 27%, mean age of patients: 72.

Malignant neoplasms (C00-C97)	✓
Neoplasms of uncertain or unknown behaviour (D37-D48)	✗
Aplastic and other anaemias (D60-D64)	✗
Obesity and other hyperalimentation (E65-E68)	✗
Disorders of lens (H25-H28)	✗
Other forms of heart disease (I30-I52)	✗
Diseases of oesophagus, stomach and duodenum (K20-K31)	✗
Other diseases of intestines (K55-K63)	✗
Disorders of gallbladder, biliary tract and pancreas (K80-K87)	✓
Other diseases of urinary system (N30-N39)	✗

Table S62: Inclusion and exclusion criteria for cluster 50. Female ratio: 46%, mean age of patients: 72.

Malignant neoplasms (C00-C97)	✓
Neoplasms of uncertain or unknown behaviour (D37-D48)	✗
Aplastic and other anaemias (D60-D64)	✓
Obesity and other hyperalimentation (E65-E68)	✗
Disorders of lens (H25-H28)	✗
Other forms of heart disease (I30-I52)	✗
Diseases of oesophagus, stomach and duodenum (K20-K31)	✗
Other diseases of intestines (K55-K63)	✗
Other diseases of urinary system (N30-N39)	✗

Table S63: Inclusion and exclusion criteria for cluster 51. Female ratio: 37%, mean age of patients: 71.

Malignant neoplasms (C00-C97)	✓
Neoplasms of uncertain or unknown behaviour (D37-D48)	✗
Obesity and other hyperalimentation (E65-E68)	✗
Disorders of lens (H25-H28)	✗
Other forms of heart disease (I30-I52)	✗
Diseases of oesophagus, stomach and duodenum (K20-K31)	✗
Other diseases of intestines (K55-K63)	✓
Other diseases of urinary system (N30-N39)	✗

Table S64: Inclusion and exclusion criteria for cluster 52. Female ratio: 40%, mean age of patients: 73.

Malignant neoplasms (C00-C97)	✓
Neoplasms of uncertain or unknown behaviour (D37-D48)	✗
Obesity and other hyperalimentation (E65-E68)	✗
Disorders of lens (H25-H28)	✓
Other forms of heart disease (I30-I52)	✗
Diseases of oesophagus, stomach and duodenum (K20-K31)	✗
Other diseases of urinary system (N30-N39)	✗

Table S65: Inclusion and exclusion criteria for cluster 53. Female ratio: 48%, mean age of patients: 77.

Malignant neoplasms (C00-C97)	✓
Neoplasms of uncertain or unknown behaviour (D37-D48)	✗
Obesity and other hyperalimentation (E65-E68)	✗
Other forms of heart disease (I30-I52)	✗
Diseases of oesophagus, stomach and duodenum (K20-K31)	✗
Other diseases of urinary system (N30-N39)	✓

Table S66: Inclusion and exclusion criteria for cluster 54. Female ratio: 52%, mean age of patients: 77.

Malignant neoplasms (C00-C97)	✓
Neoplasms of uncertain or unknown behaviour (D37-D48)	✓
Obesity and other hyperalimentation (E65-E68)	✗
Other forms of heart disease (I30-I52)	✗
Diseases of oesophagus, stomach and duodenum (K20-K31)	✗

Table S67: Inclusion and exclusion criteria for cluster 55. Female ratio: 40%, mean age of patients: 72.

Obesity and other hyperalimentation (E65-E68)	✓
Metabolic disorders (E70-E90)	✗
Mental and behavioural disorders due to psychoactive substance use (F10-F19)	✗
Episodic and paroxysmal disorders (G40-G47)	✗
Other forms of heart disease (I30-I52)	✗
Diseases of oesophagus, stomach and duodenum (K20-K31)	✗
Diseases of liver (K70-K77)	✗
Arthropathies (M00-M25)	✗
Dorsopathies (M40-M54)	✗
Noninflammatory disorders of female genital tract (N80-N98)	✗

Table S68: Inclusion and exclusion criteria for cluster 56. Female ratio: 53%, mean age of patients: 60.

Obesity and other hyperalimentation (E65-E68)	✓
Metabolic disorders (E70-E90)	✗
Mental and behavioural disorders due to psychoactive substance use (F10-F19)	✗
Episodic and paroxysmal disorders (G40-G47)	✗
Other forms of heart disease (I30-I52)	✗
Diseases of oesophagus, stomach and duodenum (K20-K31)	✗
Diseases of liver (K70-K77)	✗
Arthropathies (M00-M25)	✗
Dorsopathies (M40-M54)	✗
Noninflammatory disorders of female genital tract (N80-N98)	✓

Table S69: Inclusion and exclusion criteria for cluster 57. Female ratio: 100%, mean age of patients: 58.

Obesity and other hyperalimentation (E65-E68)	✓
Metabolic disorders (E70-E90)	✓
Mental and behavioural disorders due to psychoactive substance use (F10-F19)	✗
Episodic and paroxysmal disorders (G40-G47)	✗
Ischaemic heart diseases (I20-I25)	✗
Other forms of heart disease (I30-I52)	✗
Diseases of oesophagus, stomach and duodenum (K20-K31)	✗
Diseases of liver (K70-K77)	✗
Arthropathies (M00-M25)	✗
Dorsopathies (M40-M54)	✗

Table S70: Inclusion and exclusion criteria for cluster 58. Female ratio: 51%, mean age of patients: 61.

Obesity and other hyperalimentation (E65-E68)	✓
Metabolic disorders (E70-E90)	✓
Mental and behavioural disorders due to psychoactive substance use (F10-F19)	✗
Episodic and paroxysmal disorders (G40-G47)	✗
Ischaemic heart diseases (I20-I25)	✓
Other forms of heart disease (I30-I52)	✗
Diseases of oesophagus, stomach and duodenum (K20-K31)	✗
Diseases of liver (K70-K77)	✗
Arthropathies (M00-M25)	✗
Dorsopathies (M40-M54)	✗

Table S71: Inclusion and exclusion criteria for cluster 59. Female ratio: 39%, mean age of patients: 66.

Obesity and other hyperalimentation (E65-E68)	✓
Mental and behavioural disorders due to psychoactive substance use (F10-F19)	✗
Episodic and paroxysmal disorders (G40-G47)	✗
Other forms of heart disease (I30-I52)	✗
Diseases of oesophagus, stomach and duodenum (K20-K31)	✗
Diseases of liver (K70-K77)	✗
Arthropathies (M00-M25)	✓
Dorsopathies (M40-M54)	✗

Table S72: Inclusion and exclusion criteria for cluster 60. Female ratio: 58%, mean age of patients: 66.

Obesity and other hyperalimentation (E65-E68)	✓
Mental and behavioural disorders due to psychoactive substance use (F10-F19)	✓
Episodic and paroxysmal disorders (G40-G47)	✗
Other forms of heart disease (I30-I52)	✗
Diseases of oesophagus, stomach and duodenum (K20-K31)	✗
Diseases of liver (K70-K77)	✗
Dorsopathies (M40-M54)	✗

Table S73: Inclusion and exclusion criteria for cluster 61. Female ratio: 31%, mean age of patients: 57.

Obesity and other hyperalimentation (E65-E68)	✓
Episodic and paroxysmal disorders (G40-G47)	✓
Other forms of heart disease (I30-I52)	✗
Diseases of oesophagus, stomach and duodenum (K20-K31)	✗
Diseases of liver (K70-K77)	✗
Dorsopathies (M40-M54)	✗

Table S74: Inclusion and exclusion criteria for cluster 62. Female ratio: 35%, mean age of patients: 62.

Obesity and other hyperalimentation (E65-E68)	✓
Other forms of heart disease (I30-I52)	✗
Diseases of oesophagus, stomach and duodenum (K20-K31)	✗
Diseases of liver (K70-K77)	✓
Dorsopathies (M40-M54)	✗

Table S75: Inclusion and exclusion criteria for cluster 63. Female ratio: 44%, mean age of patients: 60.

Obesity and other hyperalimentation (E65-E68)	✓
Other forms of heart disease (I30-I52)	✗
Diseases of oesophagus, stomach and duodenum (K20-K31)	✗
Arthropathies (M00-M25)	✗
Dorsopathies (M40-M54)	✓

Table S76: Inclusion and exclusion criteria for cluster 64. Female ratio: 55%, mean age of patients: 62.

Obesity and other hyperalimentation (E65-E68)	✓
Other forms of heart disease (I30-I52)	✗
Diseases of oesophagus, stomach and duodenum (K20-K31)	✗
Arthropathies (M00-M25)	✓
Dorsopathies (M40-M54)	✓

Table S77: Inclusion and exclusion criteria for cluster 65. Female ratio: 62%, mean age of patients: 67.

Malignant neoplasms (C00-C97)	✗
Other forms of heart disease (I30-I52)	✗
Cerebrovascular diseases (I60-I69)	✗
Diseases of oesophagus, stomach and duodenum (K20-K31)	✓
Hernia (K40-K46)	✗
Other diseases of intestines (K55-K63)	✗
Diseases of liver (K70-K77)	✗
Disorders of gallbladder, biliary tract and pancreas (K80-K87)	✗
Dorsopathies (M40-M54)	✗

Table S78: Inclusion and exclusion criteria for cluster 66. Female ratio: 49%, mean age of patients: 64.

Malignant neoplasms (C00-C97)	✗
Other forms of heart disease (I30-I52)	✗
Cerebrovascular diseases (I60-I69)	✓
Diseases of oesophagus, stomach and duodenum (K20-K31)	✓
Hernia (K40-K46)	✗
Other diseases of intestines (K55-K63)	✗
Diseases of liver (K70-K77)	✗
Disorders of gallbladder, biliary tract and pancreas (K80-K87)	✗
Dorsopathies (M40-M54)	✗

Table S79: Inclusion and exclusion criteria for cluster 67. Female ratio: 47%, mean age of patients: 75.

Malignant neoplasms (C00-C97)	✓
Other forms of heart disease (I30-I52)	✗
Diseases of oesophagus, stomach and duodenum (K20-K31)	✓
Hernia (K40-K46)	✗
Other diseases of intestines (K55-K63)	✗
Diseases of liver (K70-K77)	✗
Disorders of gallbladder, biliary tract and pancreas (K80-K87)	✗
Dorsopathies (M40-M54)	✗

Table S80: Inclusion and exclusion criteria for cluster 68. Female ratio: 42%, mean age of patients: 72.

Other forms of heart disease (I30-I52)	✗
Diseases of oesophagus, stomach and duodenum (K20-K31)	✓
Hernia (K40-K46)	✗
Other diseases of intestines (K55-K63)	✗
Diseases of liver (K70-K77)	✗
Disorders of gallbladder, biliary tract and pancreas (K80-K87)	✓
Dorsopathies (M40-M54)	✗

Table S81: Inclusion and exclusion criteria for cluster 69. Female ratio: 51%, mean age of patients: 68.

Other forms of heart disease (I30-I52)	✗
Diseases of oesophagus, stomach and duodenum (K20-K31)	✓
Hernia (K40-K46)	✓
Other diseases of intestines (K55-K63)	✗
Diseases of liver (K70-K77)	✗
Dorsopathies (M40-M54)	✗

Table S82: Inclusion and exclusion criteria for cluster 70. Female ratio: 45%, mean age of patients: 67.

Other forms of heart disease (I30-I52)	✗
Diseases of oesophagus, stomach and duodenum (K20-K31)	✓
Other diseases of intestines (K55-K63)	✗
Diseases of liver (K70-K77)	✗
Dorsopathies (M40-M54)	✓

Table S83: Inclusion and exclusion criteria for cluster 71. Female ratio: 58%, mean age of patients: 68.

Mental and behavioural disorders due to psychoactive substance use (F10-F19)	✗
Other forms of heart disease (I30-I52)	✗
Diseases of oesophagus, stomach and duodenum (K20-K31)	✓
Other diseases of intestines (K55-K63)	✗
Diseases of liver (K70-K77)	✓

Table S84: Inclusion and exclusion criteria for cluster 72. Female ratio: 44%, mean age of patients: 65.

Mental and behavioural disorders due to psychoactive substance use (F10-F19)	✓
Other forms of heart disease (I30-I52)	✗
Diseases of oesophagus, stomach and duodenum (K20-K31)	✓
Other diseases of intestines (K55-K63)	✗
Diseases of liver (K70-K77)	✓

Table S85: Inclusion and exclusion criteria for cluster 73. Female ratio: 22%, mean age of patients: 59.

Other forms of heart disease (I30-I52)	✗
Diseases of oesophagus, stomach and duodenum (K20-K31)	✓
Hernia (K40-K46)	✗
Other diseases of intestines (K55-K63)	✓
Diseases of liver (K70-K77)	✗
Dorsopathies (M40-M54)	✗

Table S86: Inclusion and exclusion criteria for cluster 74. Female ratio: 48%, mean age of patients: 71.

Other forms of heart disease (I30-I52)	✗
Diseases of oesophagus, stomach and duodenum (K20-K31)	✓
Hernia (K40-K46)	✗
Other diseases of intestines (K55-K63)	✓
Diseases of liver (K70-K77)	✓
Dorsopathies (M40-M54)	✗

Table S87: Inclusion and exclusion criteria for cluster 75. Female ratio: 36%, mean age of patients: 68.

Other forms of heart disease (I30-I52)	✗
Diseases of oesophagus, stomach and duodenum (K20-K31)	✓
Hernia (K40-K46)	✗
Other diseases of intestines (K55-K63)	✓
Dorsopathies (M40-M54)	✓

Table S88: Inclusion and exclusion criteria for cluster 76. Female ratio: 55%, mean age of patients: 71.

Other forms of heart disease (I30-I52)	✗
Diseases of oesophagus, stomach and duodenum (K20-K31)	✓
Hernia (K40-K46)	✓
Other diseases of intestines (K55-K63)	✓

Table S89: Inclusion and exclusion criteria for cluster 77. Female ratio: 43%, mean age of patients: 70.

Malignant neoplasms (C00-C97)	✗
Obesity and other hyperalimentation (E65-E68)	✗
Organic, including symptomatic, mental disorders (F00-F09)	✗
Disorders of lens (H25-H28)	✗
Ischaemic heart diseases (I20-I25)	✗
Other forms of heart disease (I30-I52)	✓
Cerebrovascular diseases (I60-I69)	✗
Diseases of arteries, arterioles and capillaries (I70-I79)	✗
Influenza and pneumonia (J09-J18)	✗
Chronic lower respiratory diseases (J40-J47)	✗
Diseases of oesophagus, stomach and duodenum (K20-K31)	✗
Arthropathies (M00-M25)	✗
Renal failure (N17-N19)	✗

Table S90: Inclusion and exclusion criteria for cluster 78. Female ratio: 53%, mean age of patients: 73.

Malignant neoplasms (C00-C97)	✗
Obesity and other hyperalimentation (E65-E68)	✗
Organic, including symptomatic, mental disorders (F00-F09)	✗
Disorders of lens (H25-H28)	✗
Ischaemic heart diseases (I20-I25)	✗
Other forms of heart disease (I30-I52)	✓
Cerebrovascular diseases (I60-I69)	✗
Diseases of arteries, arterioles and capillaries (I70-I79)	✓
Influenza and pneumonia (J09-J18)	✗
Chronic lower respiratory diseases (J40-J47)	✗
Diseases of oesophagus, stomach and duodenum (K20-K31)	✗
Arthropathies (M00-M25)	✗
Renal failure (N17-N19)	✗

Table S91: Inclusion and exclusion criteria for cluster 79. Female ratio: 52%, mean age of patients: 79.

Malignant neoplasms (C00-C97)	✗
Obesity and other hyperalimentation (E65-E68)	✗
Organic, including symptomatic, mental disorders (F00-F09)	✗
Disorders of lens (H25-H28)	✗
Ischaemic heart diseases (I20-I25)	✗
Other forms of heart disease (I30-I52)	✓
Cerebrovascular diseases (I60-I69)	✗
Influenza and pneumonia (J09-J18)	✗
Chronic lower respiratory diseases (J40-J47)	✗
Diseases of oesophagus, stomach and duodenum (K20-K31)	✗
Arthropathies (M00-M25)	✓
Renal failure (N17-N19)	✗

Table S92: Inclusion and exclusion criteria for cluster 80. Female ratio: 59%, mean age of patients: 75.

Malignant neoplasms (C00-C97)	✗
Obesity and other hyperalimentation (E65-E68)	✗
Organic, including symptomatic, mental disorders (F00-F09)	✗
Disorders of lens (H25-H28)	✗
Ischaemic heart diseases (I20-I25)	✗
Other forms of heart disease (I30-I52)	✓
Cerebrovascular diseases (I60-I69)	✗
Influenza and pneumonia (J09-J18)	✓
Chronic lower respiratory diseases (J40-J47)	✗
Diseases of oesophagus, stomach and duodenum (K20-K31)	✗
Renal failure (N17-N19)	✗

Table S93: Inclusion and exclusion criteria for cluster 81. Female ratio: 55%, mean age of patients: 80.

Malignant neoplasms (C00-C97)	✗
Obesity and other hyperalimentation (E65-E68)	✗
Organic, including symptomatic, mental disorders (F00-F09)	✗
Disorders of lens (H25-H28)	✓
Ischaemic heart diseases (I20-I25)	✗
Other forms of heart disease (I30-I52)	✓
Cerebrovascular diseases (I60-I69)	✗
Chronic lower respiratory diseases (J40-J47)	✗
Diseases of oesophagus, stomach and duodenum (K20-K31)	✗
Renal failure (N17-N19)	✗

Table S94: Inclusion and exclusion criteria for cluster 82. Female ratio: 63%, mean age of patients: 80.

Malignant neoplasms (C00-C97)	✗
Obesity and other hyperalimentation (E65-E68)	✓
Organic, including symptomatic, mental disorders (F00-F09)	✗
Ischaemic heart diseases (I20-I25)	✗
Other forms of heart disease (I30-I52)	✓
Cerebrovascular diseases (I60-I69)	✗
Chronic lower respiratory diseases (J40-J47)	✗
Diseases of oesophagus, stomach and duodenum (K20-K31)	✗
Renal failure (N17-N19)	✗

Table S95: Inclusion and exclusion criteria for cluster 83. Female ratio: 51%, mean age of patients: 67.

Malignant neoplasms (C00-C97)	✓
Organic, including symptomatic, mental disorders (F00-F09)	✗
Ischaemic heart diseases (I20-I25)	✗
Other forms of heart disease (I30-I52)	✓
Cerebrovascular diseases (I60-I69)	✗
Chronic lower respiratory diseases (J40-J47)	✗
Diseases of oesophagus, stomach and duodenum (K20-K31)	✗
Renal failure (N17-N19)	✗

Table S96: Inclusion and exclusion criteria for cluster 84. Female ratio: 48%, mean age of patients: 77.

Organic, including symptomatic, mental disorders (F00-F09)	✓
Other degenerative diseases of the nervous system (G30-G32)	✗
Ischaemic heart diseases (I20-I25)	✗
Other forms of heart disease (I30-I52)	✓
Cerebrovascular diseases (I60-I69)	✗
Chronic lower respiratory diseases (J40-J47)	✗
Diseases of oesophagus, stomach and duodenum (K20-K31)	✗
Renal failure (N17-N19)	✗

Table S97: Inclusion and exclusion criteria for cluster 85. Female ratio: 70%, mean age of patients: 86.

Organic, including symptomatic, mental disorders (F00-F09)	✓
Other degenerative diseases of the nervous system (G30-G32)	✓
Ischaemic heart diseases (I20-I25)	✗
Other forms of heart disease (I30-I52)	✓
Cerebrovascular diseases (I60-I69)	✗
Chronic lower respiratory diseases (J40-J47)	✗
Diseases of oesophagus, stomach and duodenum (K20-K31)	✗
Renal failure (N17-N19)	✗

Table S98: Inclusion and exclusion criteria for cluster 86. Female ratio: 74%, mean age of patients: 86.

Ischaemic heart diseases (I20-I25)	✗
Other forms of heart disease (I30-I52)	✓
Cerebrovascular diseases (I60-I69)	✗
Chronic lower respiratory diseases (J40-J47)	✗
Diseases of oesophagus, stomach and duodenum (K20-K31)	✓
Other diseases of intestines (K55-K63)	✗
Renal failure (N17-N19)	✗

Table S99: Inclusion and exclusion criteria for cluster 87. Female ratio: 55%, mean age of patients: 74.

Ischaemic heart diseases (I20-I25)	✗
Other forms of heart disease (I30-I52)	✓
Cerebrovascular diseases (I60-I69)	✗
Chronic lower respiratory diseases (J40-J47)	✗
Diseases of oesophagus, stomach and duodenum (K20-K31)	✓
Other diseases of intestines (K55-K63)	✓
Renal failure (N17-N19)	✗

Table S100: Inclusion and exclusion criteria for cluster 88. Female ratio: 52%, mean age of patients: 75.

Obesity and other hyperalimentation (E65-E68)	✗
Metabolic disorders (E70-E90)	✗
Disorders of lens (H25-H28)	✗
Ischaemic heart diseases (I20-I25)	✓
Other forms of heart disease (I30-I52)	✓
Cerebrovascular diseases (I60-I69)	✗
Diseases of arteries, arterioles and capillaries (I70-I79)	✗
Influenza and pneumonia (J09-J18)	✗
Chronic lower respiratory diseases (J40-J47)	✗
Other diseases of intestines (K55-K63)	✗
Renal failure (N17-N19)	✗

Table S101: Inclusion and exclusion criteria for cluster 89. Female ratio: 48%, mean age of patients: 77.

Obesity and other hyperalimentation (E65-E68)	✗
Metabolic disorders (E70-E90)	✓
Disorders of lens (H25-H28)	✗
Ischaemic heart diseases (I20-I25)	✓
Other forms of heart disease (I30-I52)	✓
Cerebrovascular diseases (I60-I69)	✗
Diseases of arteries, arterioles and capillaries (I70-I79)	✗
Influenza and pneumonia (J09-J18)	✗
Chronic lower respiratory diseases (J40-J47)	✗
Other diseases of intestines (K55-K63)	✗
Renal failure (N17-N19)	✗

Table S102: Inclusion and exclusion criteria for cluster 90. Female ratio: 39%, mean age of patients: 73.

Obesity and other hyperalimentation (E65-E68)	✗
Disorders of lens (H25-H28)	✓
Ischaemic heart diseases (I20-I25)	✓
Other forms of heart disease (I30-I52)	✓
Cerebrovascular diseases (I60-I69)	✗
Diseases of arteries, arterioles and capillaries (I70-I79)	✗
Influenza and pneumonia (J09-J18)	✗
Chronic lower respiratory diseases (J40-J47)	✗
Other diseases of intestines (K55-K63)	✗
Renal failure (N17-N19)	✗

Table S103: Inclusion and exclusion criteria for cluster 91. Female ratio: 57%, mean age of patients: 80.

Obesity and other hyperalimentation (E65-E68)	✗
Ischaemic heart diseases (I20-I25)	✓
Other forms of heart disease (I30-I52)	✓
Cerebrovascular diseases (I60-I69)	✗
Diseases of arteries, arterioles and capillaries (I70-I79)	✗
Influenza and pneumonia (J09-J18)	✓
Chronic lower respiratory diseases (J40-J47)	✗
Other diseases of intestines (K55-K63)	✗
Renal failure (N17-N19)	✗

Table S104: Inclusion and exclusion criteria for cluster 92. Female ratio: 50%, mean age of patients: 81.

Obesity and other hyperalimentation (E65-E68)	✓
Ischaemic heart diseases (I20-I25)	✓
Other forms of heart disease (I30-I52)	✓
Cerebrovascular diseases (I60-I69)	✗
Diseases of arteries, arterioles and capillaries (I70-I79)	✗
Chronic lower respiratory diseases (J40-J47)	✗
Other diseases of intestines (K55-K63)	✗
Renal failure (N17-N19)	✗

Table S105: Inclusion and exclusion criteria for cluster 93. Female ratio: 41%, mean age of patients: 69.

Ischaemic heart diseases (I20-I25)	✓
Other forms of heart disease (I30-I52)	✓
Cerebrovascular diseases (I60-I69)	✗
Diseases of arteries, arterioles and capillaries (I70-I79)	✓
Chronic lower respiratory diseases (J40-J47)	✗
Other diseases of intestines (K55-K63)	✗
Renal failure (N17-N19)	✗

Table S106: Inclusion and exclusion criteria for cluster 94. Female ratio: 44%, mean age of patients: 77.

Ischaemic heart diseases (I20-I25)	✓
Other forms of heart disease (I30-I52)	✓
Cerebrovascular diseases (I60-I69)	✗
Chronic lower respiratory diseases (J40-J47)	✗
Other diseases of intestines (K55-K63)	✓
Renal failure (N17-N19)	✗

Table S107: Inclusion and exclusion criteria for cluster 95. Female ratio: 49%, mean age of patients: 78.

Obesity and other hyperalimentation (E65-E68)	✗
Other forms of heart disease (I30-I52)	✓
Cerebrovascular diseases (I60-I69)	✗
Influenza and pneumonia (J09-J18)	✗
Chronic lower respiratory diseases (J40-J47)	✓
Dorsopathies (M40-M54)	✗
Renal failure (N17-N19)	✗

Table S108: Inclusion and exclusion criteria for cluster 96. Female ratio: 42%, mean age of patients: 77.

Obesity and other hyperalimentation (E65-E68)	✓
Other forms of heart disease (I30-I52)	✓
Cerebrovascular diseases (I60-I69)	✗
Influenza and pneumonia (J09-J18)	✗
Chronic lower respiratory diseases (J40-J47)	✓
Dorsopathies (M40-M54)	✗
Renal failure (N17-N19)	✗

Table S109: Inclusion and exclusion criteria for cluster 97. Female ratio: 38%, mean age of patients: 69.

Other forms of heart disease (I30-I52)	✓
Cerebrovascular diseases (I60-I69)	✗
Influenza and pneumonia (J09-J18)	✗
Chronic lower respiratory diseases (J40-J47)	✓
Dorsopathies (M40-M54)	✓
Renal failure (N17-N19)	✗

Table S110: Inclusion and exclusion criteria for cluster 98. Female ratio: 56%, mean age of patients: 75.

Other forms of heart disease (I30-I52)	✓
Cerebrovascular diseases (I60-I69)	✗
Influenza and pneumonia (J09-J18)	✓
Chronic lower respiratory diseases (J40-J47)	✓
Other diseases of the respiratory system (J95-J99)	✗
Renal failure (N17-N19)	✗

Table S111: Inclusion and exclusion criteria for cluster 99. Female ratio: 40%, mean age of patients: 78.

Other forms of heart disease (I30-I52)	✓
Cerebrovascular diseases (I60-I69)	✗
Influenza and pneumonia (J09-J18)	✓
Chronic lower respiratory diseases (J40-J47)	✓
Other diseases of the respiratory system (J95-J99)	✓
Renal failure (N17-N19)	✗

Table S112: Inclusion and exclusion criteria for cluster 100. Female ratio: 38%, mean age of patients: 74.

Organic, including symptomatic, mental disorders (F00-F09)	✗
Episodic and paroxysmal disorders (G40-G47)	✗
Disorders of lens (H25-H28)	✗
Ischaemic heart diseases (I20-I25)	✗
Other forms of heart disease (I30-I52)	✓
Cerebrovascular diseases (I60-I69)	✓
Diseases of arteries, arterioles and capillaries (I70-I79)	✗
Renal failure (N17-N19)	✗
Other diseases of urinary system (N30-N39)	✗

Table S113: Inclusion and exclusion criteria for cluster 101. Female ratio: 53%, mean age of patients: 78.

Organic, including symptomatic, mental disorders (F00-F09)	✗
Episodic and paroxysmal disorders (G40-G47)	✗
Disorders of lens (H25-H28)	✗
Ischaemic heart diseases (I20-I25)	✓
Other forms of heart disease (I30-I52)	✓
Cerebrovascular diseases (I60-I69)	✓
Diseases of arteries, arterioles and capillaries (I70-I79)	✗
Renal failure (N17-N19)	✗
Other diseases of urinary system (N30-N39)	✗

Table S114: Inclusion and exclusion criteria for cluster 102. Female ratio: 43%, mean age of patients: 77.

Organic, including symptomatic, mental disorders (F00-F09)	✗
Episodic and paroxysmal disorders (G40-G47)	✗
Disorders of lens (H25-H28)	✓
Other forms of heart disease (I30-I52)	✓
Cerebrovascular diseases (I60-I69)	✓
Diseases of arteries, arterioles and capillaries (I70-I79)	✗
Renal failure (N17-N19)	✗
Other diseases of urinary system (N30-N39)	✗

Table S115: Inclusion and exclusion criteria for cluster 103. Female ratio: 56%, mean age of patients: 81.

Organic, including symptomatic, mental disorders (F00-F09)	✗
Episodic and paroxysmal disorders (G40-G47)	✗
Other forms of heart disease (I30-I52)	✓
Cerebrovascular diseases (I60-I69)	✓
Diseases of arteries, arterioles and capillaries (I70-I79)	✗
Renal failure (N17-N19)	✗
Other diseases of urinary system (N30-N39)	✓

Table S116: Inclusion and exclusion criteria for cluster 104. Female ratio: 73%, mean age of patients: 83.

Organic, including symptomatic, mental disorders (F00-F09)	✗
Episodic and paroxysmal disorders (G40-G47)	✓
Other forms of heart disease (I30-I52)	✓
Cerebrovascular diseases (I60-I69)	✓
Diseases of arteries, arterioles and capillaries (I70-I79)	✗
Renal failure (N17-N19)	✗

Table S117: Inclusion and exclusion criteria for cluster 105. Female ratio: 50%, mean age of patients: 78.

Organic, including symptomatic, mental disorders (F00-F09)	✗
Other forms of heart disease (I30-I52)	✓
Cerebrovascular diseases (I60-I69)	✓
Diseases of arteries, arterioles and capillaries (I70-I79)	✓
Dorsopathies (M40-M54)	✗
Renal failure (N17-N19)	✗

Table S118: Inclusion and exclusion criteria for cluster 106. Female ratio: 44%, mean age of patients: 79.

Organic, including symptomatic, mental disorders (F00-F09)	✗
Other forms of heart disease (I30-I52)	✓
Cerebrovascular diseases (I60-I69)	✓
Diseases of arteries, arterioles and capillaries (I70-I79)	✓
Dorsopathies (M40-M54)	✓
Renal failure (N17-N19)	✗

Table S119: Inclusion and exclusion criteria for cluster 107. Female ratio: 51%, mean age of patients: 79.

Organic, including symptomatic, mental disorders (F00-F09)	✓
Other degenerative diseases of the nervous system (G30-G32)	✗
Other forms of heart disease (I30-I52)	✓
Cerebrovascular diseases (I60-I69)	✓
Renal failure (N17-N19)	✗
Other diseases of urinary system (N30-N39)	✗

Table S120: Inclusion and exclusion criteria for cluster 108. Female ratio: 56%, mean age of patients: 83.

Organic, including symptomatic, mental disorders (F00-F09)	✓
Other degenerative diseases of the nervous system (G30-G32)	✗
Other forms of heart disease (I30-I52)	✓
Cerebrovascular diseases (I60-I69)	✓
Renal failure (N17-N19)	✗
Other diseases of urinary system (N30-N39)	✓

Table S121: Inclusion and exclusion criteria for cluster 109. Female ratio: 70%, mean age of patients: 85.

Organic, including symptomatic, mental disorders (F00-F09)	✓
Other degenerative diseases of the nervous system (G30-G32)	✓
Other forms of heart disease (I30-I52)	✓
Cerebrovascular diseases (I60-I69)	✓
Renal failure (N17-N19)	✗

Table S122: Inclusion and exclusion criteria for cluster 110. Female ratio: 67%, mean age of patients: 85.

Aplastic and other anaemias (D60-D64)	✗
Organic, including symptomatic, mental disorders (F00-F09)	✗
Disorders of lens (H25-H28)	✗
Other forms of heart disease (I30-I52)	✓
Diseases of arteries, arterioles and capillaries (I70-I79)	✗
Influenza and pneumonia (J09-J18)	✗
Chronic lower respiratory diseases (J40-J47)	✗
Renal failure (N17-N19)	✓
Other diseases of urinary system (N30-N39)	✗

Table S123: Inclusion and exclusion criteria for cluster 111. Female ratio: 48%, mean age of patients: 78.

Aplastic and other anaemias (D60-D64)	✗
Organic, including symptomatic, mental disorders (F00-F09)	✗
Disorders of lens (H25-H28)	✓
Other forms of heart disease (I30-I52)	✓
Diseases of arteries, arterioles and capillaries (I70-I79)	✗
Influenza and pneumonia (J09-J18)	✗
Chronic lower respiratory diseases (J40-J47)	✗
Renal failure (N17-N19)	✓
Other diseases of urinary system (N30-N39)	✗

Table S124: Inclusion and exclusion criteria for cluster 112. Female ratio: 49%, mean age of patients: 80.

Aplastic and other anaemias (D60-D64)	✗
Organic, including symptomatic, mental disorders (F00-F09)	✗
Other forms of heart disease (I30-I52)	✓
Diseases of arteries, arterioles and capillaries (I70-I79)	✗
Influenza and pneumonia (J09-J18)	✗
Chronic lower respiratory diseases (J40-J47)	✓
Renal failure (N17-N19)	✓
Other diseases of urinary system (N30-N39)	✗

Table S125: Inclusion and exclusion criteria for cluster 113. Female ratio: 41%, mean age of patients: 77.

Aplastic and other anaemias (D60-D64)	✗
Organic, including symptomatic, mental disorders (F00-F09)	✗
Other forms of heart disease (I30-I52)	✓
Diseases of arteries, arterioles and capillaries (I70-I79)	✗
Influenza and pneumonia (J09-J18)	✗
Renal failure (N17-N19)	✓
Other diseases of urinary system (N30-N39)	✓

Table S126: Inclusion and exclusion criteria for cluster 114. Female ratio: 69%, mean age of patients: 81.

Aplastic and other anaemias (D60-D64)	✗
Organic, including symptomatic, mental disorders (F00-F09)	✗
Other forms of heart disease (I30-I52)	✓
Diseases of arteries, arterioles and capillaries (I70-I79)	✗
Influenza and pneumonia (J09-J18)	✓
Chronic lower respiratory diseases (J40-J47)	✗
Renal failure (N17-N19)	✓

Table S127: Inclusion and exclusion criteria for cluster 115. Female ratio: 52%, mean age of patients: 81.

Aplastic and other anaemias (D60-D64)	✗
Organic, including symptomatic, mental disorders (F00-F09)	✗
Other forms of heart disease (I30-I52)	✓
Diseases of arteries, arterioles and capillaries (I70-I79)	✗
Influenza and pneumonia (J09-J18)	✓
Chronic lower respiratory diseases (J40-J47)	✓
Renal failure (N17-N19)	✓

Table S128: Inclusion and exclusion criteria for cluster 116. Female ratio: 45%, mean age of patients: 80.

Aplastic and other anaemias (D60-D64)	✓
Organic, including symptomatic, mental disorders (F00-F09)	✗
Other forms of heart disease (I30-I52)	✓
Diseases of arteries, arterioles and capillaries (I70-I79)	✗
Diseases of oesophagus, stomach and duodenum (K20-K31)	✗
Renal failure (N17-N19)	✓

Table S129: Inclusion and exclusion criteria for cluster 117. Female ratio: 57%, mean age of patients: 80.

Aplastic and other anaemias (D60-D64)	✗
Organic, including symptomatic, mental disorders (F00-F09)	✗
Other forms of heart disease (I30-I52)	✓
Diseases of arteries, arterioles and capillaries (I70-I79)	✓
Chronic lower respiratory diseases (J40-J47)	✗
Other disorders of the skin and subcutaneous tissue (L80-L99)	✗
Renal failure (N17-N19)	✓

Table S130: Inclusion and exclusion criteria for cluster 118. Female ratio: 46%, mean age of patients: 79.

Aplastic and other anaemias (D60-D64)	✗
Organic, including symptomatic, mental disorders (F00-F09)	✗
Other forms of heart disease (I30-I52)	✓
Diseases of arteries, arterioles and capillaries (I70-I79)	✓
Chronic lower respiratory diseases (J40-J47)	✗
Other disorders of the skin and subcutaneous tissue (L80-L99)	✓
Renal failure (N17-N19)	✓

Table S131: Inclusion and exclusion criteria for cluster 119. Female ratio: 46%, mean age of patients: 79.

Aplastic and other anaemias (D60-D64)	✗
Organic, including symptomatic, mental disorders (F00-F09)	✗
Other forms of heart disease (I30-I52)	✓
Diseases of arteries, arterioles and capillaries (I70-I79)	✓
Chronic lower respiratory diseases (J40-J47)	✓
Renal failure (N17-N19)	✓

Table S132: Inclusion and exclusion criteria for cluster 120. Female ratio: 36%, mean age of patients: 78.

Aplastic and other anaemias (D60-D64)	✓
Organic, including symptomatic, mental disorders (F00-F09)	✗
Other forms of heart disease (I30-I52)	✓
Diseases of arteries, arterioles and capillaries (I70-I79)	✓
Diseases of oesophagus, stomach and duodenum (K20-K31)	✗
Renal failure (N17-N19)	✓

Table S133: Inclusion and exclusion criteria for cluster 121. Female ratio: 49%, mean age of patients: 79.

Aplastic and other anaemias (D60-D64)	✗
Organic, including symptomatic, mental disorders (F00-F09)	✓
Other degenerative diseases of the nervous system (G30-G32)	✗
Other forms of heart disease (I30-I52)	✓
Osteopathies and chondropathies (M80-M94)	✗
Renal failure (N17-N19)	✓

Table S134: Inclusion and exclusion criteria for cluster 122. Female ratio: 55%, mean age of patients: 84.

Aplastic and other anaemias (D60-D64)	✗
Organic, including symptomatic, mental disorders (F00-F09)	✓
Other degenerative diseases of the nervous system (G30-G32)	✗
Other forms of heart disease (I30-I52)	✓
Osteopathies and chondropathies (M80-M94)	✓
Renal failure (N17-N19)	✓

Table S135: Inclusion and exclusion criteria for cluster 123. Female ratio: 81%, mean age of patients: 86.

Aplastic and other anaemias (D60-D64)	✗
Organic, including symptomatic, mental disorders (F00-F09)	✓
Other degenerative diseases of the nervous system (G30-G32)	✓
Other forms of heart disease (I30-I52)	✓
Renal failure (N17-N19)	✓

Table S136: Inclusion and exclusion criteria for cluster 124. Female ratio: 66%, mean age of patients: 86.

Aplastic and other anaemias (D60-D64)	✓
Organic, including symptomatic, mental disorders (F00-F09)	✓
Other forms of heart disease (I30-I52)	✓
Diseases of oesophagus, stomach and duodenum (K20-K31)	✗
Renal failure (N17-N19)	✓

Table S137: Inclusion and exclusion criteria for cluster 125. Female ratio: 65%, mean age of patients: 86.

Nutritional anaemias (D50-D53)	✗
Aplastic and other anaemias (D60-D64)	✓
Other forms of heart disease (I30-I52)	✓
Diseases of oesophagus, stomach and duodenum (K20-K31)	✓
Renal failure (N17-N19)	✓

Table S138: Inclusion and exclusion criteria for cluster 126. Female ratio: 51%, mean age of patients: 79.

Nutritional anaemias (D50-D53)	✓
Aplastic and other anaemias (D60-D64)	✓
Other forms of heart disease (I30-I52)	✓
Diseases of oesophagus, stomach and duodenum (K20-K31)	✓
Renal failure (N17-N19)	✓

Table S139: Inclusion and exclusion criteria for cluster 127. Female ratio: 57%, mean age of patients: 81.

REFERENCES

Haug, N., Deischinger, C., Gyimesi, M., Kautzky-Willer, A., Thurner, S., and Klimek, P. (2020). High-risk multimorbidity patterns on the road to cardiovascular mortality. *BMC Medicine* 18, 1–12. doi:10.1186/s12916-020-1508-1
